# Combining flipped-classroom and spaced-repetition learning in a master-level bioinformatics course

**DOI:** 10.1371/journal.pcbi.1012863

**Published:** 2025-04-15

**Authors:** Gabriele Pozzati, Samuel Coulbourn Flores

**Affiliations:** 1 Department of Biochemistry and Biophysics, Stockholm University, Stockholm, Sweden; 2 Department of Animal Biosciences, Swedish University of Agricultural Sciences, Uppsala, Sweden; SIB Swiss Institute of Bioinformatics, SWITZERLAND

## Abstract

Introductory bioinformatics courses can be challenging to teach. Students with a biological background may have never encountered computer science, and computer science students are likely to have minimal knowledge of biology. To improve learning, we implemented a flipped spaced-repetition course. We repeated the topics through various activities across different days while applying an unusually high number of examinations. The examinations were synergistic with the flipped classroom, encouraging reading and watching recorded lectures before in-person discussions. Additionally, they helped us structure and assess laboratory practicals. We analyzed grades, pass rates, student satisfaction, and student comments qualitatively and quantitatively over 7 years of the course, documenting progress as well as the effect of disruptions such as COVID-19 and changes in teaching staff. We share our results and insights into the opportunities and challenges of this pedagogical approach. An open online version of this course is freely provided for students and teachers.

## Author summary

A challenge of teaching Bioinformatics is that it is an interdisciplinary field which develops students’ skills in both Biology and Computer Science. We faced the additional challenge of a course that had historically had a low passing rate and poor evaluations. Of the many pedagogical techniques in current practice, flipped, and spaced-repetition learning are among the best quantified and documented. Our work explains how we implemented these two techniques with the help of Learning Management Systems and other educational technology. We quantitatively and qualitatively evaluate the results based on 7 years of grades and student evaluations. To facilitate the implementation of our method, we provide a Massively Open Online Course and cloneable Learning Management System instance, cloud-based virtual laboratory notebooks, videos, and other resources.

## Introduction

Multidisciplinary subjects such as Bioinformatics present a challenge to teachers. These courses include many ideas, methods, and applications that might be hard to understand for many students. Pedagogical techniques such as flipped-classroom and spaced-repetition learning help us face this challenge.

Flipped-classroom generally refers to a switch between the type of activities taking place inside and outside the classroom [1]. Students are required to complete passive tasks (readings, watching recorded lectures) in advance, while the lecture is replaced with active learning [2], including activities such as peer discussion and solving problems. This stimulates higher cognitive functions [3,4], provides an opportunity for insightful interactions, and helps students cross the Zone of Proximal Development—the gap between what can be learned alone and what can be learned with teacher support [5,6]. Another advantage of this system is a more efficient use of the teacher’s subject knowledge and ability to diagnose mislearned knowledge [5]. Flipped learning has grown in popularity during the last decade, as reviewed by Akçayır and colleagues [7]. This study revealed that flipped-classroom improves certain course aspects, such as learning performance, satisfaction, and engagement. On the downside, the method requires a time investment from both students and teachers. Other reports describe similarly successful applications of flipped-classroom to bioinformatics courses [8,9], confirming how question and answer, peer group discussion and problem based learning are preferred ways to engage students in class. Additionally, these studies show how clarification of course objectives, proper setting of student groupings and management of workload are essential to optimize students’ engagement.

Another outcome of pedagogical research is spaced-repetition learning, which has been shown in a large number of studies to improve retention relative to massed learning [10–12]. While massed learning refers to acquiring knowledge in a single session, spaced-repetition involves reviewing such knowledge with a certain temporal delay from the initial learning activity. In particular this method improves long-term retention of knowledge, with larger delays associated with longer recall [13]. This effect has been linked to a reduction in repetition suppression—the tendency of the brain to stop responding to frequently repeated stimuli [14,15]. A study has shown that repetition suppression lasts up to 24 h after the initial stimulus [16]. Interestingly, an old psychological study reported a peak in learning efficiency after a 24-h lag [17]—a peak reproduced in recent works [18]. Accordingly, a meta-analysis of spaced-repetition publications has shown a strong effect of 1-day delays between learning stimuli improving medium/long-term recall [13]. Nevertheless longer delays are associated with better long-term retention, practical factors like teaching period length represents an important limitation, making 1-day delays an attractive option.

Another important detail is repeated testing, which is known to improve spaced-repetition outcomes compared to repeated studying [19,20]. This means that testing should be exploited as a learning and not just an assessment tool [21–23]. Notably, while we found recent publications reporting the success of spaced-repetition in STEM and medical courses [23–25], only one mentioned the possibility to apply it to a bioinformatics-related teaching activity [26]. In a study showing a positive effect of spaced-repetition on some STEM courses, different testing instances requiring recall of the same information were applied multiple times with a delay of 2 weeks between each instance, after providing initial knowledge exposure [23]. A medical course took advantage of natural reoccurrence of topics across different course modules [25], while another adopted a program creating test flashcards on medical topics [24]. This program allows for a student-personalized testing schedule, with incremental delays depending on the student recall. A similar system of flashcards has been developed in the bioinformatic-focused study, mentioning the possibility to use this system to implement spaced-repetition [26].

The role of stress in learning must also be acknowledged and managed, to avoid negative aspects [6]. Unexpected stimuli and anxiety can be detrimental to functions important for learning, such as working memory, abstraction, and problem solving [27–29]. This argues for avoiding any aspect of the learning environment that unnecessarily increases anxiety [29].

In this study, we report the outcome of integrating these pedagogic tools and insights synergistically in a master-level bioinformatics course. Alongside grades, we collected course evaluations from 2018 to 2024 to monitor students’ opinions and obtain feedback on the course organization. We also noted the effect of the COVID-19 pandemic and a change in the course direction (as well as the natural turnover of TAs) during the reported period. This enhances the importance of this study, allowing the evaluation of the proposed interventions to highlight potential strengths and pitfalls that could otherwise have been overlooked in more optimal conditions.

## Methods

### Ethics statement

Course evaluations completed by students and used in this study can be obtained from Stockholm University. These evaluations do not identify the students who completed them. In cases where students’ textual comments provided in supplementary materials name specific teachers and TAs, the names were changed to Professor X, Professor Y, TA, etc. Supplementary data on grades and pass rates are also fully anonymized. Due to public availability and/or anonymity, our data does not require ethical approval in accordance with the Swedish Ethical Review Act and Stockholm University rules.

### Course context

The course is in the context of the SciLifeLab Molecular Techniques in Life Science (MTLS) master program, jointly given by KTH, Stockholm University, and the Karolinska Institute, which admits 20–30 students per year. Admissions require a 3-year bachelor degree, including 10 ECTS points of math and 20 ECTS of life sciences (1.5 ECTS represents 1 week of full-time study). Within the program, and before joining our course, students take 6 ECTS of communication, 6 ECTS of biostatistics, 5 ECTS of molecular genetics and genomics, and 13 ECTS of translational medicine. Our course runs full time, meaning students ideally spend 40 h per week on it and take no other courses concurrently, for about five weeks. Up to and including Spring 2022, our course also admitted non-MTLS Stockholm University students. From 2023 the course became exclusive to MTLS students.

### Course structure

Our course spans over 4 weeks and aims to teach bioinformatics fundamentals with focus on biological sequence annotation and protein structure prediction. Different topics are contained in separate modules (Table A in S1 Appendix) handled by different teachers. The first course edition in 2018 followed a traditional course structure, where each module consisted of one lecture held in the morning, followed by a laboratory in the afternoon. A written laboratory report was required weekly but the final grade was based entirely on a final exam. This 2018 edition is considered in this work as a baseline, enhanced from 2019 with our pedagogical interventions.

From the 2019 edition each module was adapted to run over a variable number of days according to the following general structure:

#### Pre-assignment.

The day before the in-person discussion, students complete an assigned reading, and watch a video on the module material.

#### Pre-discussion quiz.

Questions focused on pre-assignment material, aimed at lower levels of Bloom’s taxonomy.

#### Discussion.

In-person interactive activities are led by the module’s responsible teacher, usually in the morning. The focus is on higher levels of Bloom’s taxonomy.

#### Laboratory exercise.

TAs introduce and assist with a practical exercise on the module topic. This is done after the discussion, in the late morning/ early afternoon.

#### Laboratory quiz.

After the lab exercise, students answer a quiz that examines the module theory, verifies the performance on the exercise, and enforces reflection on the results.

**Fig 1:**
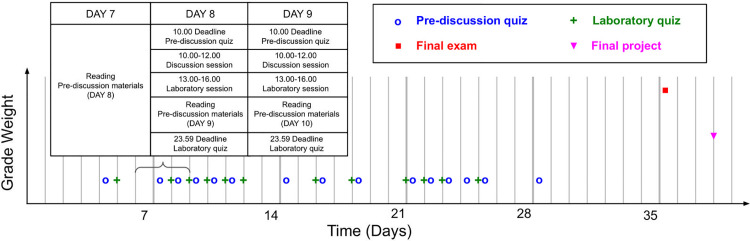
Examinations over time. Our course comprised 28 examinations over 30 course days. Most of these were semi automated pre-discussion (blue circles) and laboratories (green crosses) quizzes. This was followed by the study period, the final exam, the report writing period, and the report due date. Days 7–9 expanded as an example. Times taken from the 2024 edition of our course.

At the end of the course, there is an exam study period, then a final exam, and lastly a final project report writing period. In this work “examination” refers to any graded element. Thus any given topic is examined up to four times across the course, with variable lag in between (Fig 1).

**Fig 2:**
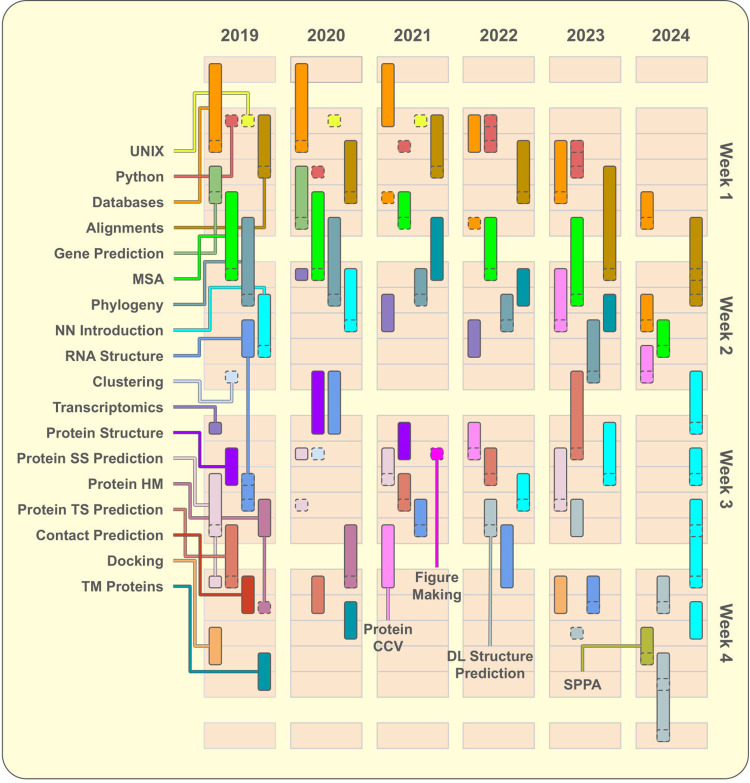
Modules organization over different course editions. Course editions starting from 2019 are represented side-by-side, while different working days and weeks of the same course edition are displayed vertically. Course edition of 2018 is omitted here due to its trivial structure, where each module appearing in 2019 occupied one day only in the same order they are listed. Individual modules have been represented as vertical bars and squares. Colors identify the same modules across all course editions. Bars’ length indicates the time dedicated to each module, while squares with dashed borders indicate when the module’s laboratory was held. Connections between labels and modules indicate which module corresponds to every color. The first day in every module (except Python, UNIX and Clustering which consisted of laboratories only) indicates scheduled pre-discussion readings. If a module including a laboratory is 2 (even nonconsecutive) days long, the discussion is always held on the same day of the laboratory, while modules longer than three days include more than one pre-discussion/discussion. A short module description is provided in Table A in S1 Appendix; Abbreviations follow. MSA, Multiple Sequence Alignments; NN, Neural Networks; DL, Deep Learning; SS, Secondary Structure; HM, Homology Modelling; TS, Tertiary Structure; TM, TransMembrane; CCV, Classification, Comparison and Visualization; SPPA, Spatial Point Pattern Analysis.

Course modules have undergone significant evolution during reported course editions (Fig 2). Many modules, such as contact prediction, homology modeling or UNIX, have been aggregated or removed to reduce the workload. Some other modules, such as Neural Network Introduction have been extended, to match new trends. Module organization has also been affected by variations in examination schemes and logistical necessities. One example is the adoption of a partial exam from 2019 to 2022, which forced us to interrupt classrooms to give students some time to prepare for the mid-course examination. Details about all interventions are reported in the Methods section and summarized in Table 1.

**Table 1:** Summary of interventions on course organization.

Course edition	Presence	Laboratory	Programming platform	Exam type	Partial exams	Open book
2018	In person	Desktop	Unix	Paper	No	No
2019	In person	Desktop	Unix	Canvas	Yes	No
2020	In person	Mixed	Unix	Canvas	Yes	No
2021	Remote	Laptops	Unix	Canvas	Yes	Yes
2022	Hybrid	Laptops	CoLab	Exam.net	Yes	Yes
2023	In person	Laptops	CoLab	Paper	No	Yes
2024	In person	Laptops	CoLab	Paper	No	Yes

### Pre-discussion assignments

Assignments include book chapters and prerecorded video lectures. Student’s preparation before class is evaluated and enforced by mandatory, automatically graded pre-discussion quizzes. These quizzes are not intended to be difficult or stressful, but to test basic understanding of the module material. They are typically composed of a small number of simple automatically-graded questions, and are due before the start of the discussion. A few examples of pre-discussion quiz questions are reported in Table B in S1 Appendix. Students are given three attempts at the quiz (with the last score prevailing), and the automatically graded score is given each time, so they have a chance to improve their answers (in practice students usually reach 80%–100%). Pre-discussion quizzes collectively give 10% of the final course grade.

### Classroom discussions

Many formats have been used for the in-person discussion across different modules. Discussions usually took place in a classroom, but during the Covid-19 pandemic we were forced to adapt our format to online classes (Table 1).

One common activity requires the teacher to create groups of 3–5 students and assign them a few discussion topics to discuss (for 10–15 min) in more depth than in the pre-discussion quiz. Conclusions are then shared by each group with the whole class, stimulating questions from peers and comments from the teacher. This activity was particularly convenient during 2021 and 2022 editions, when discussions were run remotely on Zoom (https://zoom.us/) taking advantage of breakout rooms for sub-group discussions. Problem based learning was also included in some module’s group work. Solving a conceptually difficult problem required active elaboration of module concepts, and stimulated participation as well as social interactions. Some examples include applying information theory to assess a language translator, using Bayes’ theorem to test common social stereotypes, or formulating a problem encoding to be solved by machine learning models.

Depending on the module, discussions could also be more similar to a traditional lecture, where topic explanations were interspersed with question and answer sessions. The Mentimeter interactive presentation system (https://www.mentimeter.com/) was particularly suitable for this purpose. This platform enabled structured discussions alternating lecturer’s slides to surveys whose answers can be screened and quantified precisely. An additional advantage of this system is the student’s ability to answer anonymously, stimulating participation of more introverted individuals. Some concepts were best explained with physical models, for example a knotted rope to illustrate phylogenetic trees, or plastic molecules to demonstrate dihedral angles and steric clashes.

### Laboratories

Most modules’ discussions are followed by sessions of practical exercises in the afternoon. Laboratories allow students to work with bioinformatics data, tools and databases. Laboratory exercises were created and/or refined by one TA who was assigned each year to each module. Where possible, exercises included some programming or command line related aspect. TAs were also responsible for providing short (10 min) introductions at the beginning of the laboratory sessions and tutoring students through exercises.

In the first course editions, laboratories ran in a room with 30 desktop computers. In 2020, we reduced the use of fixed desktop computer laboratories in favor of student’s private laptops, together with a dedicated CPU server accessible through remote connection. Desktops were completely abandoned in 2021, during COVID-19 pandemic lockdown and afterwards. From 2023, the dedicated server was replaced by free cloud services such as Google CoLab (https://colab.google/).

Laboratory examinations have also changed significantly from those of 2018 editions. In our baseline course, each week of afternoon practice was followed by the production of a laboratory report, while other course editions replaced these reports with near-daily learning platform quizzes testing the understanding of the practical exercises. Such quizzes are also mandatory graded elements, posing generally harder questions compared to pre-discussion quizzes. Three attempts are allowed, as is collaboration. Correct answers were made available after the laboratory quiz deadline. Examples of questions posed in these quizzes are available in Table B in S1 Appendix.

### Final report

Starting in 2019, a long-format report was required at the end of the course. During course editions from 2019 to 2023, the report consisted of analyzing a protein sequence for which students should determine phylogenetics, 3D structure, function and other properties. In 2024 the final report consisted of creating a Deep Learning (DL) model for protein secondary structure prediction. In both cases, a common project format and a list of tasks containing several mandatory and optional points were provided to students as a guide to follow in compiling their report. Two afternoon sessions in every course edition were dedicated to provide further guidance for student projects.

### Final exam

For the final exam, we used different formats, always containing the same type of essay questions, to adapt to different course settings (Table 1). In the 2018, 2023 and 2024 editions the exam was on paper, while from 2019 to 2022 it was digital. From 2019 to 2021 the exam was split into two partial exams hosted on Canvas. In 2021, due to lockdown restrictions, we held this exam remotely, with the students connected on Zoom, keeping their videos and microphones active. In 2022 partial examinations were implemented on the exam.net platform (https://exam.net/). In 2021 and 2022, due to remote examinations, we allowed students free access to notes, course books, and internet resources during examination. In 2023 and 2024 only notes were allowed, due to the exam being on paper. All exam answers were checked for plagiarism through the Urkund system. Several activity-free days were set aside for study before each exam. Examples of prior years’ exams have been made available to the students and are available in S6 Appendix.

### Course grading

In 2018 we based 100% of the grade on the final exam, and the students wrote weekly laboratory reports which were not graded, but required for course credit. Since 2019, the final grade for the course has then been composed of: pre-discussion quizzes (10% of the final grade), laboratory quizzes (10%), final report (30%), and final examination (50%). Each final exam question was graded by the teacher responsible for the related module. Each report was graded between 0 and 100 by two TAs who evaluated format (20% of the final report grade), completeness (40%), critical thinking (20%) and figures (20%). If the two grades had standard deviation above 5, a teacher was added as a third grader.

At Stockholm University, grading criteria are announced at the beginning of the course. In 2018 (and prior years) the pass line was 50 (of 100), because the traditional approach was resulting in low grades. In 2019 we raised our pass line to 60, consistent with the aim of improving learning outcomes. A grade of 55–59 is assigned a failing grade of “Fx”; the teacher can assign a task to demonstrate mastery of weak areas (“Komplettering” in Swedish) and thus raise to a “E,” the lowest passing grade. According to the Swedish Higher Education Ordinance, students have the right to retake any exam, if they fail both the exam and the overall course. Reexamination opportunities are offered twice yearly.

### Learning platform

Starting in 2019, the course’s Learning Management System has been the free version of Canvas (https://www.instructure.com/canvas). On this platform, the preparation, discussion materials and quizzes are grouped in the appropriate modules. Canvas supports creation and grading of quizzes with automatic (e.g., numeric, multiple choice) and manual (e.g., essay, image upload, file upload) graded question types. It also allows for activity scheduling, participant discussions, file storage and provides email notifications for relevant activity.

### Data analysis

We computed effect size of adopted interventions by comparing final exam grades of each course edition with those from the baseline edition using CohenD with pooled standard deviation [30]:


$$d=\frac{{\bar{x}}_{1}-{\bar{x}}_{b}}{s}$$

$$s=\sqrt{\frac{\left(n_{1}-1\right)s_{1}^{2}-\left(n_{b}-1\right)s_{b}^{2}}{n_{1}+n_{b}-2}}$$

with *x*, *n*, and *s*^2^ indicating grades average, number of students, and grades standard deviations, respectively. Values related to 2018 course editions are represented by the *b* subscript, while the 1 indicates numbers referred to any other edition.

Course evaluations are applied by the Chemical Section at the Faculty of Science of Stockholm University. Some questions are set by the Chemistry Section, but these can change from year to year, creating challenges in extracting and analyzing data. Another challenge was to not over-represent individual students’ opinions in the analysis of free text comments. Anonymous answers pooled under each question forced us to analyze separately the results of individual questions to enable a simple quantification of different positive and negative aspects in students’ perception. We selected *“What did you like in the course?”* and *“Which improvements would you suggest for the course?”* to summarize a qualitative evaluation of all course editions. Answers to these questions were analyzed following the protocol proposed by Dierckx de Casterlé and colleagues [31]. All plotting and data analysis has been performed on a Google CoLab notebook with the default Python libraries Matplotlib and Numpy. The CoLab notebook used for data analysis and students’ comments categorizations are provided in S2 Appendix.

## Results and discussion

### Grades and pass rates

**Fig 3:**
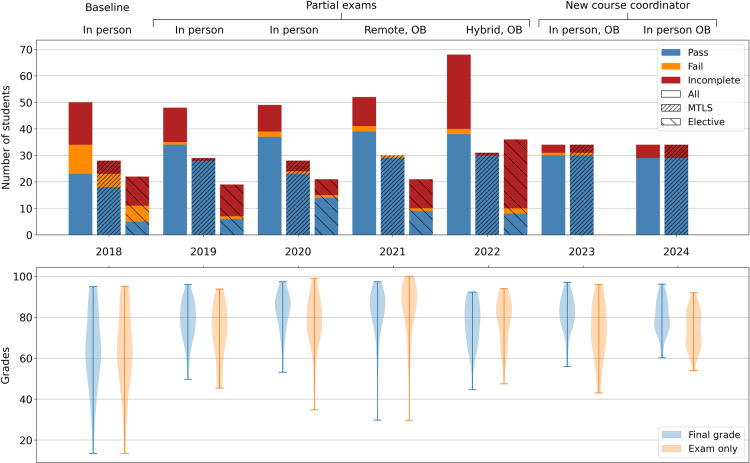
Students’ outcomes and grade distributions. The top panel plot shows variation of course outcomes for the Bioinformatic course between 2018 and 2024; Each bar represents the number of students who passed the course by completing all mandatory elements and getting a final grade equal or higher than the passing threshold (blue), the number of those who failed (orange) and the number of those who did not complete all required elements (red). Striped bars show data for students who chose this course as an optional element (sparse stripes) and MTLS master students (dense stripes). Non-striped bars summarize the same data for all enrolled students, starting from 2018. The bottom panel shows the distribution of final grades (blue) and exam grades (orange) over different course editions for students who completed the course. In course editions using partial exams, the combination of the two votes is reported. Labels on top of the plots indicate secondary course interventions. OB stands for open book exam.

Grades and pass rates are perhaps the simplest measures of learning outcomes. Some of the students participating in this course have to take it as a mandatory element in the SciLifeLab Molecular Techniques in Life Science master program (MTLS group), while other students took it as an elective (elective group). It is clear that most students dropping the course were from the elective group (Fig 3, top panel). This could be due to the more selective nature of the MTLS program, to the higher variability in backgrounds of elective students or simply to the fact that the elective group had the option of choosing other courses. Due to the low pass rate, we decided in 2023 to stop offering our course to elective students. The subsequent increase in the ratio of “Incomplete” MTLS students in 2023 and 2024, indicates that the fraction of students completing the course is not dependent on the overall number of enrolled students. Similarly, while the number of students in the elective group completing the course increased to different extents compared to 2019, these numbers do not correlate with the total number of enrolled students. In 2022 this is particularly striking given that the number of enrolled students greatly increased. A possible explanation for this could be our choice to make the 2022 course edition to run hybrid (meaning mixed in-person and remote) due to the COVID-19 pandemic. The lower ratio of passing students might be due to a disadvantage of online students compared to in-person students, or to the difficulty of coordinating hybrid teaching. Looking at the overall proportions over different years, up to 2021 passing students kept increasing, while the total number of enrolled students remained approximately constant (Fig 3, top panel). It is interesting to note that 2021 results were slightly better than 2020, suggesting that in-person and 100% remote teaching can have similar outcomes. To exclude a possible confounding effect of students’ backgrounds on these observations, we retrieved available data on MTLS students bachelor studies. From this, it is clear that MTLS students mostly have a biological or other scientific background; a smaller proportion have medical, engineering, and humanities backgrounds (Fig A in S1 Appendix). We found no strong difference in grades between these groups, though engineering (not including biotechnology) students had marginally lower average grades. This indicates that observed fluctuations in background composition of groups of students does not explain year-to-year fluctuations in grades.

In line with previous studies [9], we argue that our final exam was similar in goals, format, and level from year to year and thus allows for comparison between different years’ interventions and our 2018 baseline. It is useful to look at the distribution of grades through all course editions (Fig 3, bottom panel), in order to evaluate which set of interventions provided the best learning outcome. Comparing exam grades of 2019 and 2020 editions with 2018 editions results in medium (CohenD: 0.49) and large (CohenD: 0.8) effect sizes respectively. The comparison between 2021 and 2018 grades produces an even larger effect size (CohenD: 1.17), while the distributions of exam grades for the hybrid course of 2022, and for the in-person editions of 2023 and 2024 also show medium-large effect sizes (CohenD: 0.87, 0.51 and 0.6 respectively). Overall, this data provides evidence about the effectiveness of the proposed interventions, in terms of student performance. Our implementation of flipped-classroom and spaced-repetition has shown improvements in 2019 and 2020 relying only on small modules’ adjustments and partial exams as auxiliary pedagogical interventions. The largest effect was observable in 2021 when the course was fully remote due to COVID-19 pandemic and open book examination was subsequently forced by circumstances. Additional interventions were proven in later editions to not be sufficient (open-book in 2021) nor necessary (partial exams in 2023 and 2024) for large results improvements compared to 2018 edition, highlighting the necessity of a good course structure. It is also clear from these data how integrating a large number of graded elements (quizzes and project scores) in the final score partially mitigates bad exam results while making it harder to obtain a perfect overall score.

### Students’ satisfaction

**Fig 4:**
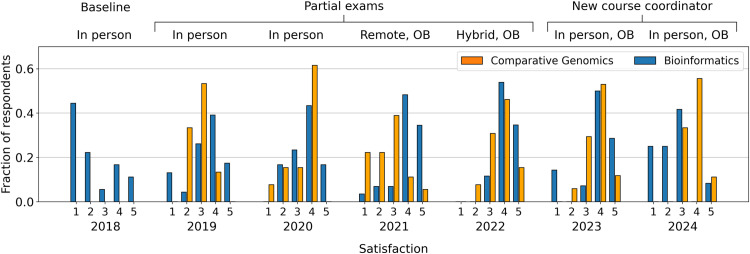
Agreement with the statement “I’m satisfied with the course”. Satisfaction categories are indicated on the horizontal axis for all reported course editions, and vary from 1 (“Not at all)” to 5 (“Completely”), while the vertical axis reports the fraction of students falling in each category. Data is collected from course evaluations of two successive courses (Bioinformatics and Comparative Genomics) of the MTLS programme over different years to ensure that variations we observe are not cohort-specific. Labels on top of the plots indicate secondary course interventions. OB stands for open book exam.

Satisfaction was measured in all course evaluations and has the advantage of not being biased by grading criteria. Comparison of average grade vs. average satisfaction shows only outlier-driven correlation (Fig B in S1 Appendix), meaning that this metric should be considered, on top of grades, to obtain complementary insights on course outcomes. Survey results on student satisfaction match the behavior of grade distributions from 2018 to 2021 (Fig 4). Satisfaction peaks in 2022, the last year Flores was course coordinator. It then decreases slightly in 2023 and strongly in 2024, thus diverging from grades behavior. We discuss this in detail in the section “Students’ feedback: 2023/2024”. This data has been compared with the satisfaction for Comparative Genomics, a course which is also flipped, computational, and required for MTLS students. From the comparison, it is shown that variations we observe between different years are not cohort-specific, due to major discrepancies between satisfaction for the two courses in 2021 and 2024.

### Students’ feedback: 2018 baseline

To gain a better understanding of students’ engagement in different course editions, we selected two course evaluation questions, those asking students’ opinions on the best course aspects and possible improvements. Coding of the related answers according to Dierckx de Casterlé and colleagues [31] provided a summary of Suggested Improvements (SI) and Favoured Elements (FE) that can be adopted to illustrate pros and cons of adopted interventions (Tables 2 and 3). One example is the 2018 edition, where SI indicate that students were exhausted by the excessive workload, due to the extensive (ungraded) lab reports and poor lecturer skill (preparation, enunciation) (Table 2). The combination of these issues with the poor social skills of teaching assistants and problematic scheduling, impaired the learning outcomes of this course edition. Regardless of these problems, students have mostly selected as FE the topics presented in the course, as well as programming notions (Table 3).

**Table 2:** Coding of students’ answers to the question “*Which improvements would you suggest for the course?*”.

Code (Category)	Description	2018 (*n* = 19)	2019 ( *n* = 23)	2020 ( *n* = 30)	2021 ( *n* = 29)	2022 ( *n* = 26)	2023 ( *n* = 14)	2024 ( *n* = 12)	Example
Readings (Workload)	Amount of pre-discussion readings	0	**10** ^**^	4	0	0	0	0	“[…] Also, do not assign more than 50 pages of reading in an evening. […]”
Content (Workload)	Amount of topics	4	4	4	0	0	2	1	[…]Some of the lectures seemed over-packed with information.
Activities (Workload)	Amount of tasks to complete	**7** ^**^	3	1	0	0	**4** ^**^	2	The quizzes everyday were quite stressful […]
Time (Workload)	Requirements for more time	1	**5** ^*^	**9** ^**^	**7** ^**^	0	1	**4** ^*^	Make it a period A-B course […]
Videos (Teaching)	Quality issues in pre-discussion videos	0	0	0	1	**11** ^**^	2	3	[…] videos had a poor quality sound and it was hard to understand the lecturer.
Topics (Teaching)	Topic difficulty or relevance	2	3	1	1	1	1	1	Broaden aspects of bioinformatics as this was primarily based on structural bioinformatics.
Depth (Teaching)	Necessity of higher detail or focus in explanations	2	0	2	2	4	**3** ^*^	1	[…] It is sometimes very unclear what information is more important to carry with us.
Skill (Teaching)	Teachers’ pedagogical skill	**7** ^**^	4	**9** ^**^	**4** ^*^	**5** ^*^	**3** ^*^	3	[…] X’s lectures were hard to follow.
Coordination (Organization)	Information and logistical support	2	1	1	**4** ^*^	0	1	2	Better organization, especially in the beginning […]
Restructuring (Organization)	Alternative scheduling or methodologies	**5** ^*^	3	2	**4** ^*^	3	1	**5** ^**^	Change the time schedule, giving some break between the lecture and lab. […]
Materials (Organization)	Quality/availability of book and slides	2	2	1	2	4	1	1	The book for reading is from years ago. we need something new
Format (Examinations)	Formatting rules of project, exam or quizzes	1	2	2	3	3	1	3	The word limit for the secret sequence exercise was way too small. […]
Content (Examinations)	Subject of project, exam or quiz questions	0	4	**5** ^*^	0	4	1	**4** ^*^	[…] I sometimes could not answer the questions without additional reading […]
Grading (Examinations)	Grade contributions and policies	3	1	2	1	2	1	1	[…] Make clear grading scheme and instructions for the written exam.
Quality (Laboratories)	Hardness or typology of laboratory activities	2	4	3	1	1	1	1	[…]The labs were not well organized.[…]
Assistants (Laboratories)	Amount and quality of TAs support	**5** ^*^	2	1	0	0	0	0	[…] the TAs do not help you at all, just tell you to google things […]
Programming (Laboratories)	Appropriateness of programming exercises	0	2	3	**4** ^*^	0	1	1	[…] Have more time for programming introduction.

Values refer to the number of students who commented about a certain category in a certain year; Individual student answers often referred to multiple categories, hence the sum of all column values is higher than the number of students answering the survey (indicated in parentheses below the year).

** Highest number of the column.

* Second highest number in the column.

**Table 3:** Coding of students’ answers to the question “*What did you like in the course?*”.

Code (Category)	Description	2018 (*n* = 19)	2019 ( *n* = 23)	2020 ( *n* = 30)	2021 ( *n* = 29)	2022 ( *n* = 26)	2023 ( *n* = 14)	2024 ( *n* = 12)	Example
(Workload)	Time to acquired knowledge ratio	2	5	2	3	3	3	0	[…] it forces one to learn fast, and I felt that I really learned a lot.
Topics (Teaching)	Appreciation of one or more topics	**6** ^*^	**6** ^*^	2	5	3	3	**8** ^**^	[…] the neural network part is difficult but necessary […]
Skill (Teaching)	Teachers’ pedagogical skill	3	3	5	0	2	**5** ^**^	**5** ^*^	Y lectures were really clear and interesting.
Structure (Organization)	Course management and methodologies	1	**6** ^*^	6	**10** ^*^	**10** ^*^	2	0	The inverted classroom was really great! I also really liked that we came across the topics several times. […]
Materials (Organization)	Quality/availability of book, slides and video	1	4	5	2	3	2	2	[…] I liked that the lectures were recorded so we could study at our own pace. […]
(Examinations)	Formatting rules of project, exam or quizzes	0	5	6	5	3	2	1	[…] The best part of the course was the secret sequence report […]
Quality (Laboratories)	Appreciation of laboratory activities	3	**12** ^**^	**16** ^**^	**11** ^**^	**13** ^**^	**4** ^*^	3	[…] The labs were a really great learning experience. […]
Assistants (Laboratories)	Amount and quality of TAs support	1	3	**8** ^*^	7	4	1	1	[…]All assistants in the lab were really helpful and good at their job.
Programming (Laboratories)	Appreciation of programming exercises	**9** ^**^	1	3	2	1	1	0	Learning bash and python will be very useful for me in the future.[…]

Values refer to the number of students who commented about a certain category in a certain year; Individual student answers often referred to multiple categories, hence the sum of all column values is higher than the number of students answering the survey (indicated in parentheses below the year).

** Highest number of the column.

* Second highest number in the column.

### Students’ feedback: 2019

**Fig 5:**
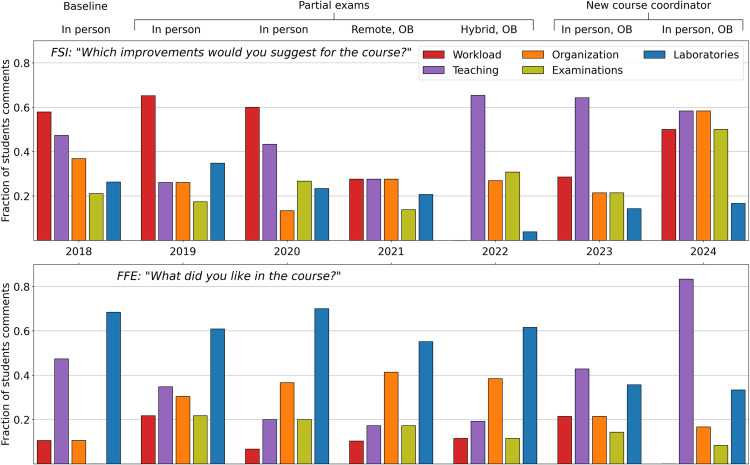
Quantification of improvement and preference comments. Students’ free-text answers have been coded according to the procedure proposed by Dierckx de Casterlé and colleagues [31]. Coded answers have been joined in one or more of five categories: Workload, Teaching, Organization, Examinations, and Laboratories. Codes indicating an excessive amount of work have been included in the Workload category. Codes about course topics, quality of teaching, and provided videos were collected in the Teaching category. Codes about course management, coordination, and other didactic materials were grouped in the Organization category. Codes about pre-lecture quizzes, final project, final examinations, and the overall grading system are in the Examination category. Finally, codes about the quality of practicals, teaching assistants’ work, and programming exercises are contained in the Laboratories category. Each bar represents for different course editions the fraction of students who gave an answer falling into a specific category, respective to the total number of students answering that year’s evaluation. Notice that a single student answer (unlimited in number of characters) could refer to multiple classes, hence the sum of fractions for different classes in the same year is always larger than 1. The top panel reports answers to the question *“What improvements would you suggest in the course?”*. The bottom panel reports answers to the question *”What was the best aspect of the course?”.*Labels on top of the plots indicate secondary course interventions. OB stands for open book exam.

Feedbacks from 2019 course editions reflect some inexperience in handling selected pedagogical interventions for the first time but also show the associated benefits. SI focused on workload, mostly addressing the excessive amount of reading materials but also the necessity of more time to run such a course and the excessive number of topics and activities. Overall, the Fraction of Suggested Improvements (FSI) about workload was only slightly higher compared to 2018 (Fig 5, top panel). Laboratories were also criticized more, due to disorganization and lack of proper introductions, but also appreciated as the best way to learn. Students assigned the highest Fraction of Favored Elements (FFE) to the laboratories category, shifting the FE from programming to general laboratories in comparison to 2018 (Fig 5, bottom panel, and Table 3).

This is likely related to the improvement of TA training to emphasize helpfulness and approachability. The new TAs were not mentioned often in the FE but were rated very high in the dedicated course evaluation question (Fig D in S1 Appendix). FFE also increased for, organization, examinations, and workload due to several students picking as FE the proposed pedagogical interventions, the final project, and the volume of learned material (Fig 5, bottom panel, and Table 3). The course organization was the topic of a separate question in the course evaluation, which shows a marked increase in scores compared to 2018 (Fig C in S1 Appendix). This confirms the positive effect of flipped classroom and repeated examinations to improve students’ active participation and allow them to slowly build up their knowledge with a much lower level of anxiety.

### Students’ feedback: 2020

Interventions on course editions in 2020 were mostly aimed to reduce the course workload, by reducing the number of modules (Fig 2) and readings. The docking module was abandoned and contact prediction was merged into the tertiary structure prediction module. The SI about excessive readings decreased, but the workload FSI was still too high due to insufficient time to properly digest course material. Teaching FSI was also high, with many SI regarding lecturer enunciation and preparation (Fig 5, top panel, and Table 2). Laboratories, course organization and TAs on the other hand, appeared to be improving (Table 3 and Figs C and D in S1 Appendix).

### Students’ feedback: 2021/2022

In 2021 and 2022, we made aggressive interventions on course modules to reduce the workload further and improve course organization. We were also facing the additional complications of dealing with the pandemic, meaning we went to remote and hybrid teaching in 2021 and 2022, and made the exams open-book and open-internet.

In 2021 Protein Homology Modelling was removed, and two modules, neural network introduction (NNIntro) and gene prediction were replaced with the protein classification, comparison, and visualization (ProteinCCV) module and a short laboratory on figure making. Such replacement aimed at a particular rearrangement of the course modules in two thematic blocks: the first focused on sequence annotation and the second focused on protein and RNA structures. These interventions leveled FSI, so that no more than 30% of students complained about any category, (Fig 5, top panel), while the highest FFE remained assigned to laboratories and course organization (Fig 5 bottom panel and Table 3). This indicates a better balance of different course aspects and a particular suitability of our pedagogical interventions to remote classes.

In 2022, the focus of the second half of the course switched to the (then) newly-released AlphaFold2 [32]. The protein secondary structure prediction module was deleted and the protein structure module was aggregated into the ProteinCCV module, and shortened to make room for NNIntro and a new DL for structure-prediction module. Additionally, the UNIX and figure-making laboratories were abandoned, reducing the number of modules to 12. This reduction in modules appears to be responsible for reducing the Workload FSI to zero. This was further highlighted by the self-reported weekly work hours which reached the most balanced distribution with an average investment of 45 h (Fig E in S1 Appendix). Laboratories FSI also went to almost zero (Fig 5, top panel). The likely reason is that Python labs were moved from the UNIX command line to user-friendly cloud-based programming notebooks. Students’ complaints were then redirected to videos (bad enunciation, too fast pace, too short, scattered information, etc.) for the second half of the course.

Considering the presented evidence, together with the high grades obtained by the students in these editions, we argue that the combination of lowered complaints and higher grades marks 2021 and 2022 as the most optimized versions of our course.

### Students’ feedback: 2023/2024

Course edition of 2023 marked a change as Flores left as course coordinator, and a new one was appointed. The end of the pandemic also marked the return to in-person teaching. The partial exam was dropped, and a Docking module was added. Workload FSI increased. Teaching SI were now not only about video quality but also teacher skills and the need for more focus on certain topics. Interestingly, Teaching also comprised a higher proportion of FFE, while for the first time laboratory FFE dropped below 0.5, reflecting the increase of workload SI (specifically, about the number of activities). Additionally, there was markedly less satisfaction with TAs (Fig D in S1 Appendix) possibly due to a stop in the TA training that started in 2019.

In 2024 two new textbooks were adopted: Magnus Ekman’s “Learning Deep Learning” and Jonathan Pevsner’s very extensive “Bioinformatics and Functional Genomics.” The NNIntro and (to a lesser degree) DL structure prediction modules were expanded and a new spatial point pattern analysis was added. To make room for this, the docking, python, phylogeny, RNA structure, transmembrane proteins, and secondary and tertiary structure prediction modules were dropped. This large rearrangement of scope led to the rise of FSI for all categories except laboratories. The main focus of SI concerned course organization, for example too much was expected from the at-home portion of the flipped classroom, the final project had insufficient instructions, the quizzes had errors, and the exam had irrelevant questions (Fig 5 and Table 2). This is corroborated in a separate question about course organization (Fig C in S1 Appendix). Students also complained about a high workload, specifically not having enough time to absorb materials. On the upside, the NNIntro module is credited for increasing Teaching FFE to above 0.8.

## Conclusion

The combination of repeated testing with spaced-repetition and flipped-classroom presented a great degree of success, together with challenges. The course showed increases in grades and student satisfaction. Annual evaluations have also shown a good reception of this setting, even when its implementation has been revealed to be suboptimal under several aspects. Many details have to be considered when planning a course on this model. There is a large investment of time required to produce high-quality materials and activities for pre-assignments, discussions, and practicals. Additionally, strict management of each course element timing is advised, in order to control variations in workload that can result in deleterious effects on students’ learning. The choice of this course model implies a long-term investment that might require a few iterations between different course editions to find the ideal solutions for each teaching style and subject. With this in mind, large changes should be implemented carefully and with consideration of the student experience.

### Course information

One can enroll in the Massively Open Online version of our Canvas Course using the self-signup link: https://canvas.instructure.com/enroll/Y3XEEC

Teachers should contact us to clone the course instance; videos, Google CoLab notebooks, quizzes, and other materials are available under the Creative Commons 3.0 license.

All course evaluations adopted in this study can be obtained by contacting the chemistry section’s office at Stockholm University.

### Key points

- We implemented flipped-classroom together with spaced-repetition, enforced using automated quizzes. We thus jumped from a single examination in 2018 to many (28) in 2024.
- Grades and student satisfaction improved greatly in 2019 and gradually in most years thereafter.
- We present a quantitative analysis of free-text student surveys, including opinions on organization, lectures, laboratories, examinations, and workload.
- Results provide a novel roadmap for integrating several pedagogical techniques in a Bioinformatics or similar course, using current educational technology.

## Supporting information

S1 Appendix Document containing supplementary figures and tables, including captions. (DOCX)

S2 Appendix Data analysis calculations, in Jupyter notebook format. (IPYNB)

S3 Appendix Student grades for all course editions. (XLSX)

S4 Appendix Categorization of students comments on “Suggested improvements”. (DOCX)

S5 Appendix Categorization of students comments on “Favoured elements”. (DOCX)

S6 Appendix Course final report sample with format “Secondary structure prediction”. (PDF)

S7 Appendix Final exam sample. (DOCX)
