# Supplementary material for: Combining flipped-classroom and spaced-repetition learning in a master-level bioinformatics course
Source: PLoS Comput Biol. 2025 Apr 15;21(4):e1012863. doi: 10.1371/journal.pcbi.1012863 (PMC11999146; doi:10.1371/journal.pcbi.1012863)
Supplement: S1 Appendix — (DOCX) [file pcbi.1012863.s001.docx]

Supplementary Figures & Tables


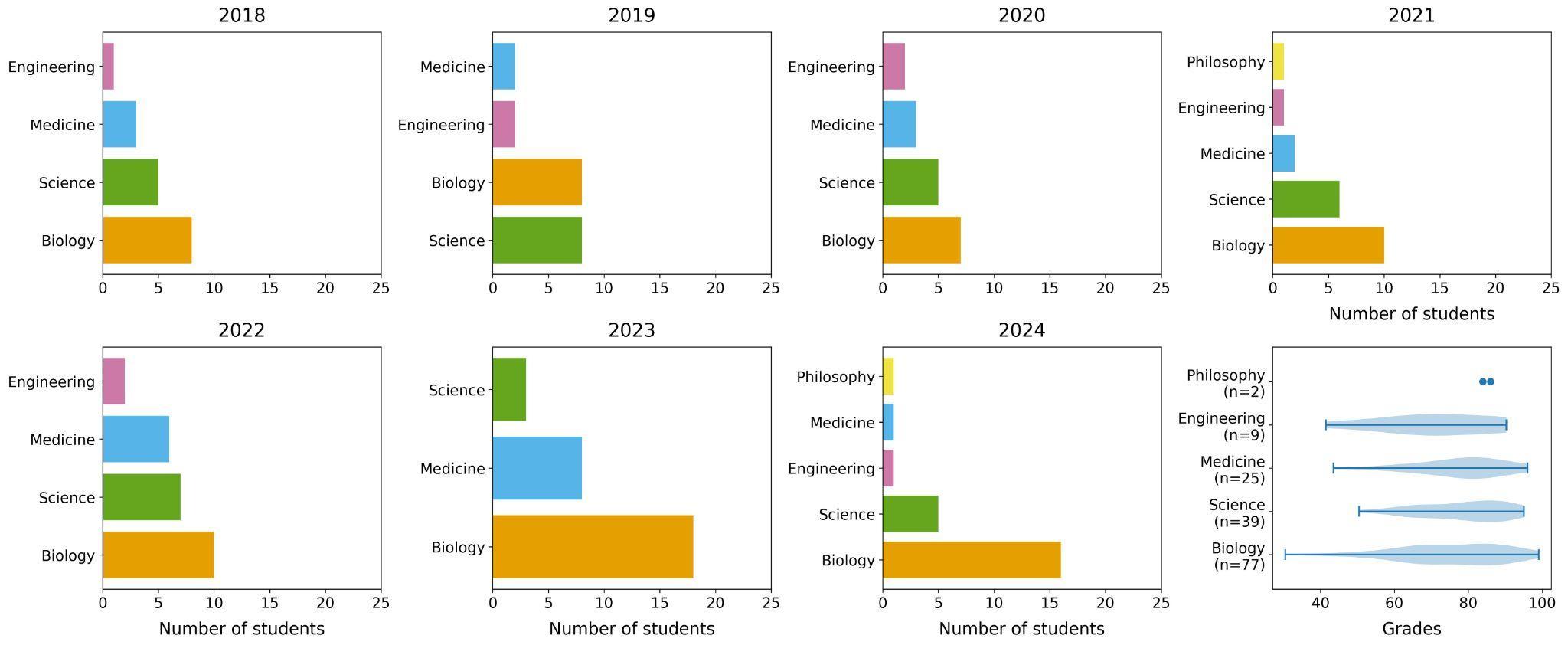


Fig A. MTLS students' backgrounds. Every individual bar plot shows the distribution of MTLS students' backgrounds for a single course edition. Bachelors with the “medic” keyword are grouped under the Medicine category. The Biology category includes all remaining scientific backgrounds including the keywords “bio”, “gen”, “pharmacy” and “agriculture”. The Science category includes remaining unlabelled Bachelor of Science degrees, plus those of Chemistry and Geology. Remaining bachelors of engineering and technology are grouped together with bachelors in Math, under the Engineering category. The last two bachelors which are not included in any of the previous categories are both Bachelors in Philosophy, and are thus indicated with that name. The violin plot shows all course editions background data plotted against the related exam grades obtained by the student in the Bioinformatics course. Due to the small amount of data points belonging to the Philosophy category, individual points have been shown instead.


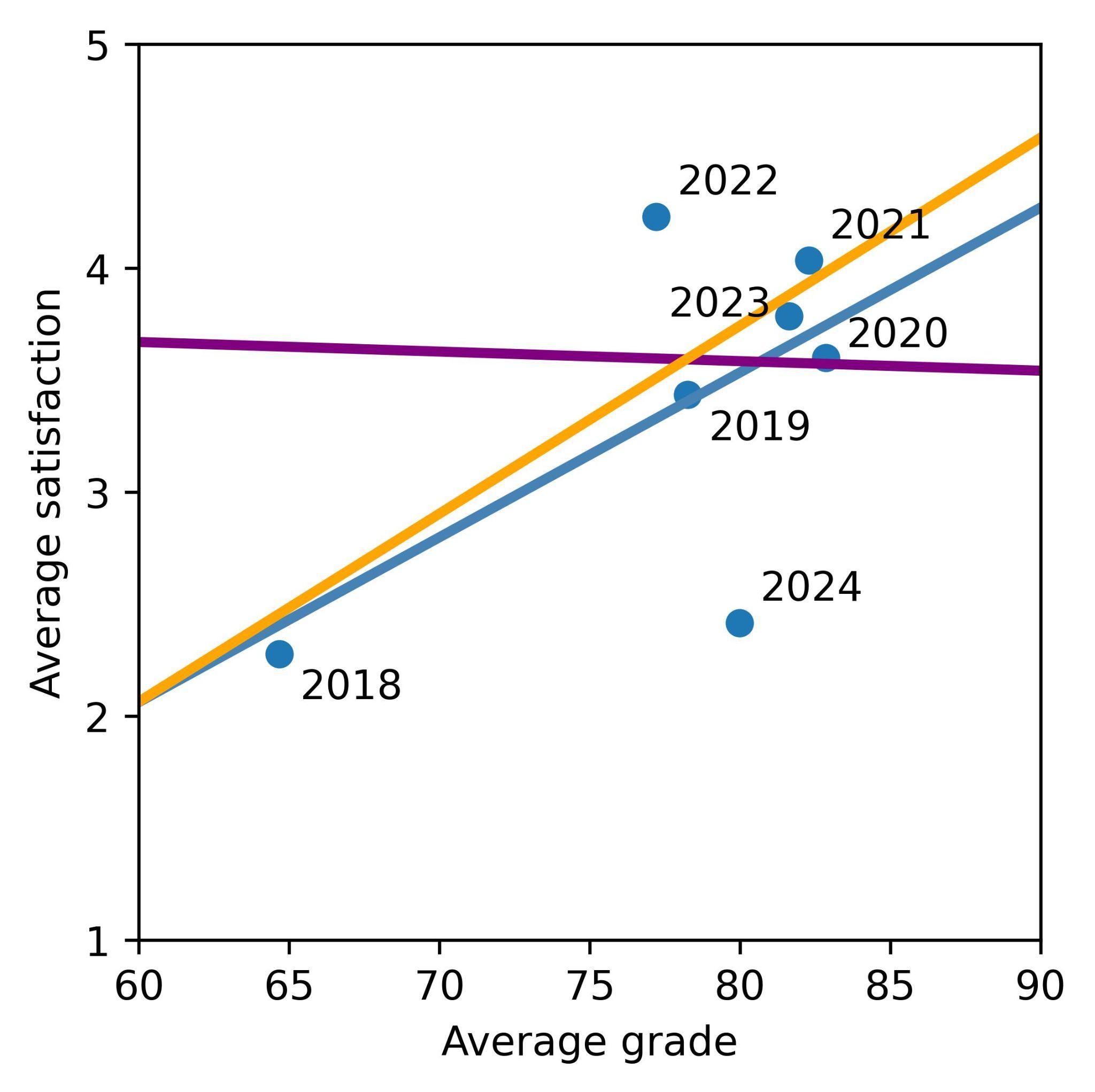


Fig B. Grades vs student satisfaction. Grades increased compared to 2018 (blue line), but correlation lacked statistical significance (PCC=0.61, p=0.15). Removing the 2018 data point makes the relationship between grades and satisfaction even less significant (purple line, PCC=-0.02, p=0.98). Removing instead the 2024 data point, the correlation becomes stronger (orange line, PCC=0.83, p=0.04).


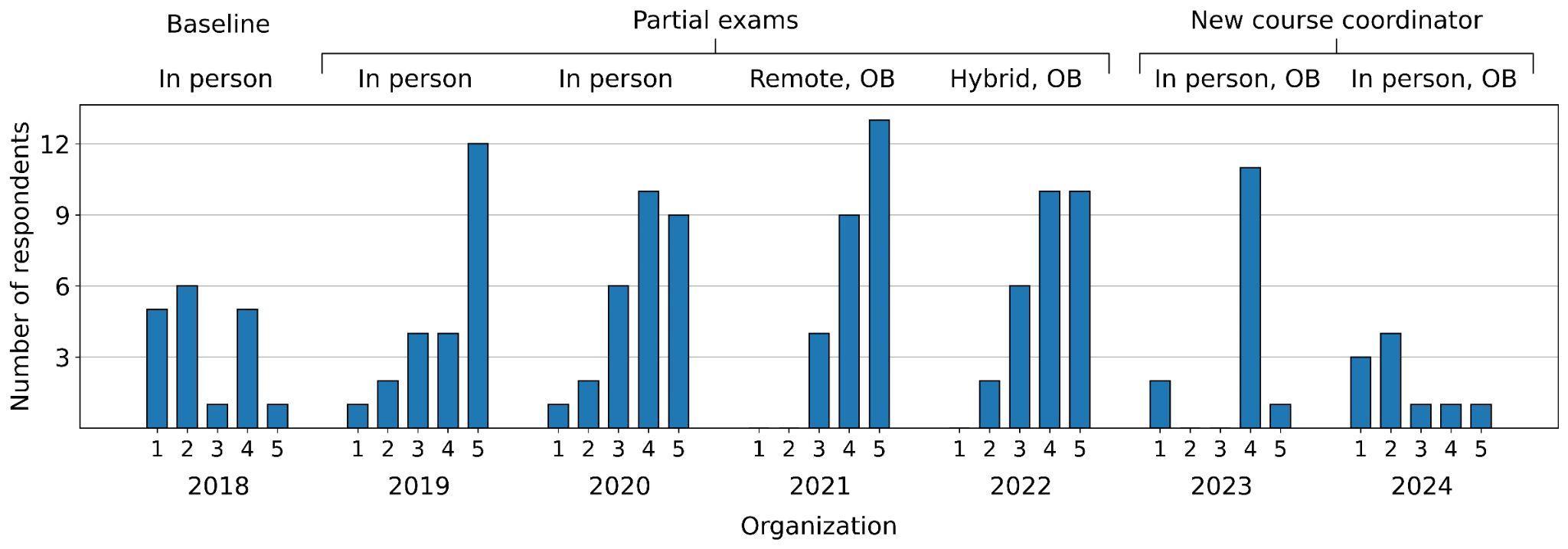
Fig C. Students’ opinion on course organization. Summary of students’ answers ranging from 1 (Completely Disagree) to 5 (Completely Agree) to different formulations of the same statement. In 2018 the statement was *“I consider the course as well structured and organized”*, while in 2019 and 2020 the statement was *“I felt that the course was well organized”*. Finally, from 2022 to 2024 the statement was *“I felt that the course was well structured and organized”*. Labels on top of the plots indicate secondary course interventions. OB stands for open book exam.


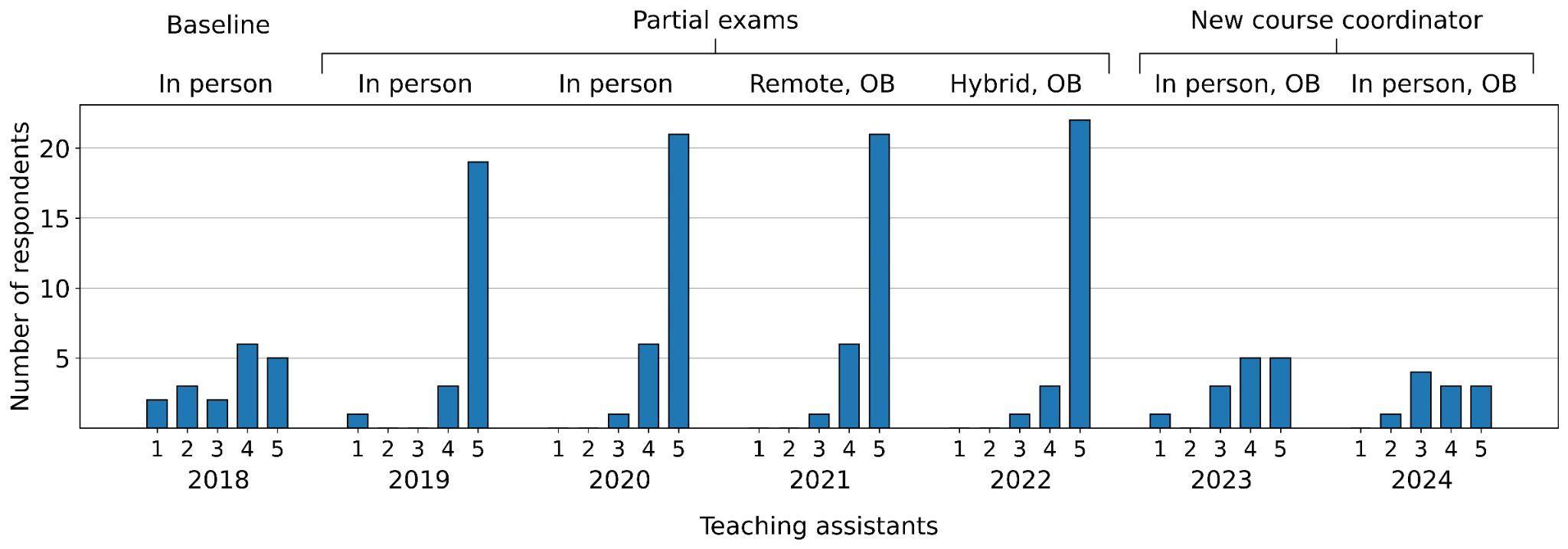
Fig D. Approval of TAs work. Students provided an answer ranging from 1 (Completely Disagree) to 5 (Completely Agree) to different formulations of the same statement. In 2018 the statement was *“I am satisfied with the lab assistant”*, while in 2019 and 2020 the statement was *“The TAs helped me achieve the course aims”*. Finally, from 2022 to 2024 the statement was *“I think the teacher assistants (if any) were good”*. Labels on top of the plots indicate secondary course interventions. OB stands for open book exam.


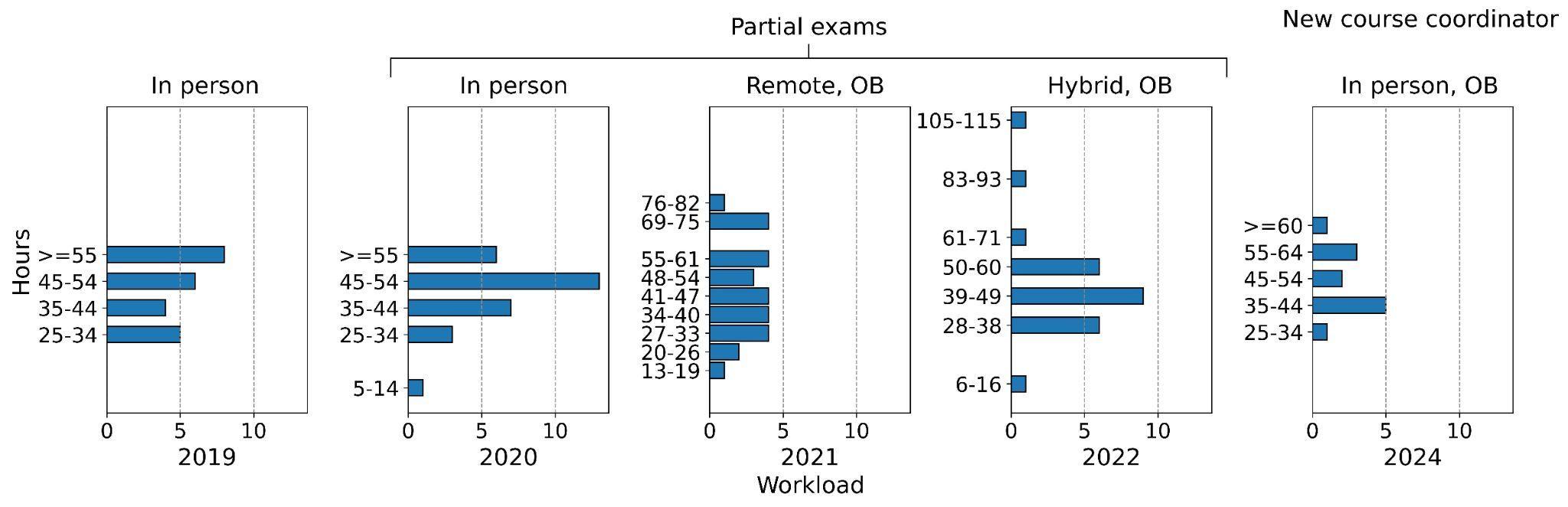


Fig E. Total weekly study hours. Horizontal axis shows the number of students falling in certain workload ranges, indicated with each bar on the vertical axis. In 2018 study hours were collected separately for theoretical and practical portions of the course, making a comparison impossible. Data from 2023 was not interpretable due to an error in the evaluation questionnaire. In the remaining years hours categories varied, so the vertical scale has been made proportional between different panels for ease of comparison. Labels on top of the plots indicate secondary course interventions. OB stands for open book exam.

Table A. Description of modules taught in the Bioinformatics course.

| **Module** | **Description** |
| --- | --- |
| UNIX | Basic knowledge of UNIX terminals, allowing file management as well as setup and use of basic programs and bioinformatic tools. |
| Python | Programming language elements, enabling production of simple programs to manipulate protein sequence and structure files. |
| Databases | Introduction of essential biological databases such as NCBI and Uniprot as well as usage of advanced search filterings, together with critical thinking over entries properties. In 2024 included protein family related databases (such as PFAM) previously covered in the “TM proteins” module. |
| Alignments | Basic sequence alignment algorithms as well as heuristic algorithms to search large sequence databases. Also focuses on metrics to evaluate alignment quality. |
| Gene Prediction | Features of genes across different kingdoms of life and how to exploit these features to identify them in genomes. |
| MSA | Algorithms to align multiple sequences in a single alignment, using these alignments to compute position specific scoring matrices or updating scoring systems to increase sensibility to remote homologs. |
| Phylogeny | Common algorithms, such as Neighbor Joining, adopted to produce phylogenetic trees, to enable comparison of sequences to evaluate the evolutionary history of a specific gene product. |
| NN Introduction | Introduction to Neural Networks programming to solve simple classification problems using a dataset of labeled examples. |
| RNA Structure | Introduction to RNA structures, usage of Macro Molecule Builder (MMB) to build simple models, rigidify domains and predict RNA structures by homology modeling. |
| Clustering | Common clustering algorithms to evaluate a distance metric between biological sequences and group them by similarity. |
| Transcriptomics | General features of transcriptomes, gene expression regulation mechanisms and experiment conditions modulation to produce annotations in metagenomic analyses. |
| Protein Structure | General features and physico-chemical properties of protein structures. |
| Protein SS Prediction | Feature of amino acid sequences enabling prediction of secondary structure elements in proteins together with main methods, such as GOR and Neural Networks, to classify them. |
| Protein HM | Introduction to the usage of homologous structures to model unknown proteins starting from sequence, using the MODELLER software. |
| Protein TS Prediction | Theoretical introduction on the use of x-ray crystallography, to solve experimentally protein structures. Definition of protein domains, together with the introduction and usage of related databases such as SCOP, CATH and ECOD. |
| Contact Prediction | Methods to predict protein structures without relying on homology. Included fragment assembly with ROSETTA and more recent contact prediction methods based on Direct Coupling Analysis and Neural Networks. |
| Docking | Introduction to the use of MMB to model protein-protein docking based on homologous interactions used as templates. |
| TM Proteins | Use of sequence profiles in HMM to represent protein families, the PFAM database and their application to identify transmembrane proteins. |
| Protein CCV | Introduction to protein structural motifs, geometrical features, common operation and styles adopted to represent and compare protein structures in visualization programs such as Pymol. |
| Figure Making | Use of Jalview and Pymol to properly visualize multiple sequence alignments and protein structures respectively, finally producing high-quality pictures. |
| DL Structure Prediction | Introduction and testing of state of the art deep learning structure prediction methods such as Alphafold2, ESM-fold and RF-diffusion. |
| SPPA | Introduction to the techniques to analyze spatial point patterns, to obtain information on protein distributions from microscopy data. |

Table B. Questions’ examples for quizzes and exams in the Bioinformatics course

PD refers to pre-discussion quizzes questions, LAB for laboratory quizzes questions and EX for final exams questions. Some of the questions refer to pictures not reported in this table. For more examples please refer to the MOOC course provided in the “Acknowledgements and Availability” section.

| **Module** | **Quiz** | **Question** | **Possible Answers** |
| --- | --- | --- | --- |
| Multiple Sequence Alignments | PD | What is a guide tree? | a) A random-forest method for building MSAs;  b) The Ancient Ones would consult it prior to a voyage;  c) A decision tree algorithm;  d) The final multiple sequence alignment;  e) A phylogenetic tree used for prioritizing pairwise sequence alignments;  f) Sort of like a spirit animal, except it's a vegetable; |
|  | LAB | Sort the results of your search by sequence identity and look at the bottom of your list. What is the organism of the lowest identity hit added to your results in the last iteration between the following ones? | a) Human Coronavirus NL63;  b) Halobact. Salinarum R1;  c) Avian bronchitis virus;  d) Homo Sapiens; |
|  | EX | Describe how ClustalW (and other progressive multiple sequence alignment methods) works. | - |
| Protein structure | PD | In homology modeling, why are loops often modeled after one is finished modeling the core? Check all that apply. | a) The gold standard data is less complete for loops;  b) The core conformation is harder to model;  c) Loops are relatively easy to attach once the core is defined;  d) The loops are less conserved in evolution;  e) Loops are thermally more mobile;  f) The core has better defined pairwise residue-residue interactions;  g) No need to invoke science here. It's just what good people do; |
|  | LAB | Build the models for these 3 templates. Check the models and discuss: what could be the reasons for the differences of the models? | a) Sequence coverage;  b) GMQE;  c) Oligo state;  d) Experimental methods; |
|  | EX | How many degrees of freedom (e.g., angles) do you need to describe the backbone conformation of a protein chain of length N residues? What amino acids does this Ramachandran plot represent? | - |
