# Supplementary material for: Combining flipped-classroom and spaced-repetition learning in a master-level bioinformatics course
Source: PLoS Comput Biol. 2025 Apr 15;21(4):e1012863. doi: 10.1371/journal.pcbi.1012863 (PMC11999146; doi:10.1371/journal.pcbi.1012863)
Supplement: S4 Appendix — (DOCX) [file pcbi.1012863.s004.docx]

QUAGOL STAGE2: narrative reports

2018:

**Excessive Workload and Unrealistic Deadlines:** Many students expressed frustration with the high intensity of the course, citing an overwhelming workload that left little time for understanding the material. Suggestions included reducing the number of assignments, labs, and lectures or extending the course duration to allow for deeper learning.

**Incomprehensible Lectures:** Numerous comments criticized the main lecturer's poor English articulation and lack of clarity, making lectures difficult to follow. Students felt this significantly hindered their ability to learn and proposed replacing the lecturer or adding support like translators or improved videos.

**Poor Course Organization:** The sequence of lectures and labs was described as inefficient, with labs often preceding the necessary theoretical background. This led to confusion and wasted time. Students recommended restructuring the course to ensure better alignment between labs and lectures.

**Inadequate Support from TAs and Professors:** Many students criticized TAs for being unhelpful, disrespectful, or unprepared. Similarly, lecturers were seen as unorganized, with slides and content appearing hastily put together. Improved preparation and more supportive, student-centered teaching approaches were suggested.

**Pace and Content Overload:** Students found the pace too fast and the volume of material overwhelming. They recommended reducing content, focusing more on conceptual understanding, and spreading topics over a longer period.

**Stressful Assessment Practices:** Several students pointed out that rigid grading rules, such as penalizing late lab submissions heavily, increased stress. They proposed more flexible deadlines and clearer grading criteria aligned with the syllabus.

**Introductory Level Misalignment:** Given the course’s introductory nature, students felt it assumed too much prior knowledge, particularly in programming. They recommended starting with more foundational teaching and gradually building up to complex tasks.

**Unprofessional Behavior:** A few students highlighted inappropriate remarks from TAs and lack of adherence to professional and respectful conduct in class. Suggestions included training for TAs on social and pedagogical skills.

**Plagiarism Accusations and Conflicting Instructions:** There was confusion and frustration about plagiarism policies, particularly when students felt they were following guidance to research online. Clarifying policies and providing better-defined tasks were suggested to avoid such conflicts.

**Scheduling Issues:** Some students noted that lecture and lab timings were suboptimal, with afternoon shifts affecting productivity. They proposed more morning sessions and fewer overlapping deadlines.

2019:

**Excessive Workload and Unrealistic Expectations:** The course workload was frequently described as overwhelming, with unrealistic expectations for daily study hours and reading assignments. Many students suggested reducing the content volume or extending the course duration to improve feasibility and learning outcomes.

**Time Management and Course Structure:** Many students found the tight scheduling problematic, with insufficient time to digest content, complete assignments, and prepare for exams. Suggestions included better distribution of readings, quizzes, and lab sessions, as well as providing more time for projects and assignments.

**Reading Assignments:** Reading loads were consistently criticized as being too heavy, with unclear relevance to quizzes and lectures. Students recommended assigning fewer pages, focusing on specific sections, and providing clearer instructions on what is essential.

**Assessment Design:** Exams and quizzes were seen as overly focused on memorization rather than understanding or application. Many suggested incorporating broader, more critical-thinking-based questions or practical applications, such as coding tasks or bioinformatics case studies.

**Lab Organization:** Labs were often described as disorganized or misaligned with the course’s bioinformatics focus. Students recommended clearer introductions to software and tools, better time allocation, and a shift toward hands-on learning over theory.

**Course Content Balance and Clarity:** Some felt that the course content lacked focus or clarity, especially in the second half. Simplifying lectures, emphasizing relevant topics, and providing better flow between topics were suggested improvements.

**Stress and Well-Being:** The intense pace and workload were described as detrimental to students’ mental and physical health. Calls for rest periods and more realistic scheduling were common.

**Group Work and Collaboration:** A few students suggested incorporating group work to enhance peer learning and reduce individual workload.

**Teacher and Resource Quality:** Issues with teaching clarity, disorganized materials, and inaccessible resources were noted. Improving teacher preparation and providing smaller, more manageable resource files were recommended.

**Relevance and Inclusivity:** Some students felt the course catered more to programmers learning biology than vice versa. Suggestions included offering foundational programming lectures to accommodate diverse student backgrounds.

2020:

**High Workload and Time Constraints**: Many students emphasized that the course had an overwhelming workload, making it difficult to process information, prepare for quizzes, or study in depth. This was especially challenging for those with limited computer science backgrounds. Requests for reduced reading material, less intensive quizzes, and more time for labs or deeper exploration of topics were common.

**Course Organization and Structure**: Numerous comments pointed to uneven distribution of topics between exams, unclear schedules, or disorganized lecture content. Some suggested making the course a 15-credit one or spreading it across two periods to alleviate the time crunch.

**Lecturer Preparation and Delivery**: Students frequently criticized the lack of preparation, clarity, and engagement in lectures. Several comments singled out specific lecturers for being unorganized or failing to communicate effectively, particularly in the first part of the course.

**Quizzes and Grading Concerns**: Many students found the pre-lecture quizzes overly time-consuming, poorly aligned with lecture content, or unhelpful for learning. They also mentioned issues with the grading system and fairness in exams, such as cheating risks in computer-based assessments and unclear expectations for math-heavy questions.

**Lab Experience**: While labs were often described as interesting and beneficial for active learning, several students felt they were too long, difficult, or overly reliant on theory. More focus on programming, increased deadlines for lab quizzes, and better preparation from lab assistants were suggested.

**Focus and Depth**: There were calls for covering fewer topics in greater depth to improve understanding, particularly for non-bioinformatics students. Students felt that too much emphasis was placed on topics like machine learning at the expense of foundational bioinformatics tools.

**Accessibility and Support for Non-Native Speakers**: Non-native English speakers reported struggling with fast-paced lectures, unclear explanations, and difficulties following along. Better materials, slower pacing, and improved use of visual aids during lectures were recommended.

**Exam Content and Preparation**: Several students expressed dissatisfaction with the exam focus, feeling it did not adequately reflect the course’s key content. Suggestions included better alignment between labs, quizzes, and exams and clearer communication of exam expectations.

**Technical and Logistical Issues**: Problems with camera angles, whiteboard views, and software used during lectures were mentioned. Improvements in lecture recordings and interactive features were suggested.

**Suggestions for Broader Improvements**: Some students advocated for emphasizing bioinformatics applications more relevant to biology-focused students, limiting reliance on theoretical content, and ensuring all educators have adequate teaching training.

2021:

**Workload and Scheduling**: Many students found the course overly intense, particularly during the first week. They suggested spreading the workload more evenly and providing course materials, such as pre-lecture content, earlier to allow better preparation.

**Teaching Style and Clarity**: Several students found some lectures and explanations unclear, advocating for more concise, structured, and straightforward teaching, as well as improved supplementary materials like slides with notes.

**Lab Structure and Support**: The lab sessions were perceived as rushed and inadequately connected to real-world applications. Students requested more time for labs, collaborative opportunities with diverse groups, and improved guidance for beginners.

**Python and Unix Training**: A recurring theme was insufficient preparation for programming tasks, especially Python. Suggestions included dedicated beginner-level Python lectures, more time for foundational exercises, and better integration of graphics and step-by-step instructions.

**Inclusivity and Group Dynamics**: Concerns were raised about group dynamics, with calls for gender-balanced groups and strategies to foster a more inclusive and collaborative environment.

**Course Scope and Content Balance**: Students suggested reevaluating the balance of topics, such as increasing focus on genomics/transcriptomics or neural networks while potentially reducing structural informatics.

**Assessment and Feedback**: Some students found the word limit for reports restrictive and expressed dissatisfaction with delays in grading and feedback on their submissions.

**Customization and Engagement**: Suggestions included allowing students to select personalized project sequences to enhance engagement and reduce individual workload by encouraging teamwork.

2022:

**Poor Quality and Clarity of Pre-Recorded Lectures**: A significant number of students criticized a teacher pre-recorded lectures for being difficult to follow due to unclear speech, poor audio quality, and insufficient structure. They recommended re-recording these lectures with clearer explanations, better slides, and subtitles. Many also suggested combining short videos into longer, more coherent sessions.

**Need for Improved Exam Clarity and Preparation**: Students found the exam questions unclear and suggested better alignment between lectures, the exam, and grading criteria. They requested more focused pre-exam sessions to review critical topics and clarify expectations.

**Outdated or Insufficient Course Material**: The course book was deemed outdated, with students advocating for a modern and relevant textbook. They also pointed out discrepancies between lecture content and required knowledge, leaving them uncertain about what to prioritize in their studies.

**Discussion Structure and Interaction**: Several students found the breakout sessions ineffective and preferred more interactive main-session discussions focusing on difficult or critical topics.

**Challenging Topics for Beginners**: Topics such as machine learning and AlphaFold were described as too advanced or fast-paced for students with a biology background. Suggestions included beginner-friendly material and more general introductions to complex concepts.

**Lab and Assignment Design**: While the labs were enjoyable, some students suggested adding more self-directed tasks and providing greater flexibility in report requirements.

**Lecture Delivery Preferences**: There were mixed opinions on using pre-recorded lectures versus live sessions, with some advocating for a clear, singular approach.

**Specific Module Feedback**: The machine learning and AlphaFold modules were described as particularly difficult to comprehend due to disorganized material and poor lecture quality.

**Technical and Legal Concerns**: Students raised concerns about the legality of requiring cameras and microphones during exams and the use of exam.net.

2023:

**Workload and Course Structure**: Many students found the course overly intense, with too much material, quizzes, and deadlines packed into a short timeframe. This left little room to digest the content properly and resulted in stress and late-night work.

**Quality and Accessibility of Learning Materials**: Students mentioned issues with outdated or unclear course materials, including the course book and PowerPoint presentations. Additionally, the quality of recorded lectures was uneven, sometimes necessitating extra effort to supplement learning.

**Clarity and Guidance**: Several students felt there was insufficient guidance, especially for self-taught components and complex topics like machine learning and AlphaFold. They suggested clearer summaries, better communication of expectations, and posting learning outcomes for each module.

**Teaching and Communication**: Some students pointed out disparities in teaching quality between lecturers and suggested assigning more lectures to effective educators. Others emphasized the need for better communication between instructors and students.

**Examinations and Grading**: Concerns were raised about the fairness of exams and harsh grading, particularly for tasks like the secret sequence exercise. Students felt underprepared for certain assessments due to insufficient instruction.

**Technical Challenges**: Navigating tools like PyMOL and organizing content on the learning platform (Canvas) were noted as difficulties. Suggestions included better organization of course materials and clearer labeling of quizzes and lectures.

**Teaching Format**: Flipped classroom approaches and the reliance on videos were criticized by some as ineffective compared to traditional teaching. Students expressed a desire for more interactive, instructor-led sessions.

**Miscellaneous Feedback**: Specific suggestions included reducing the word limit for assignments, extending deadlines, and incorporating more updated bioinformatics topics in the curriculum.

2024:

**Flipped Classroom Issues**: Several students found the flipped classroom approach ineffective, citing that the lecture videos were poorly executed (e.g., unorganized, difficult to understand, or lacking essential details). There were concerns about the lack of real lectures and the over-reliance on slides or self-study, which led to confusion and frustration. The pre-discussion quizzes were also criticized for being excessive and not particularly helpful.

**Course Structure and Workload**: Many students felt the course content was overly broad and dense for the time allocated, with some suggesting a focus either on machine learning or the theory/protein aspects. Several also noted that the workload, including quizzes and labs, was excessive, with too many deadlines and too much content to absorb in one week. The suggestion to reduce the number of lectures and leave more time for self-study was common.

**Lab and Assignment Design**: There were complaints about labs taking longer than scheduled, quizzes containing errors, and assignments being unclear or difficult due to poorly defined expectations. Some students proposed that labs should be more focused on learning rather than assessment and that assignments should come with more guidance.

**Exam and Assessment Format**: Multiple students criticized the exam for not assessing understanding deeply, instead focusing on rote memorization. They suggested making the exam more reflective of students' comprehension and adjusting the exam format to ensure it evaluated core concepts effectively. Some felt that the exam should be held earlier, to allow more time for assignments.

**Support and Resources**: A few students suggested improvements in terms of providing more help sessions for the programming project, as some students felt they were unprepared for the skills required. There were also calls for clearer guidance in assignments, and a request for feedback on marks lost in assignments to understand mistakes.

**General Teaching Approach**: There were also calls for more traditional lectures or clear video explanations, with some students emphasizing the need for scripts or additional materials to clarify complex topics.

**Pacing and Flexibility**: Some students suggested adjusting deadlines for quizzes and assignments, allowing more flexibility for students to work at their own pace. A few also recommended reducing the frequency of quizzes to decrease pressure on students.

**Student Health and Well-being**: A couple of students raised concerns about the intense schedule, advocating for at least one day off per week and a better balance between study and rest to maintain health and well-being.

QUAGOL STAGE7: coding

1 Workload

1.1 Readings (excessive amount of pre-discussion reading)

1.2 Content amount (excessive amount of topics)

1.3 Number of activities (excessive amount of tasks to complete)

1.4 Time requirement (necessity of more time)

2 Teaching

2.1 Videos (quality issues in pre-discussion videos)

2.2 Topics (issues with specific topics)

2.3 Depth (necessity of higher detail in explanations)

2.4 Pedagogical skill (individual teachers skill)

3 Course organization

3.1 Coordination (information and logistical support)

3.2 Restructuring (alterations of scheduling and methods)

3.3 Didactic materials (quality and availability of book, slides and similar)

4 Examinations

4.1 Format (issues with examination format details)

4.2 Content (issues with examined concepts)

4.3 Grading (issues with examination evaluations)

5 Laboratories

5.1 Quality (hardness or type of laboratory activities)

5.2 TA Support (amount and appropriateness of TA interactions)

5.3 Programming (accessibility of programming)

**Course Evaluation 2018**

Number of credits for this course is too low with respect to amount of material covered. **(1.2)**

1. teacher should pay more attention to his accent; **(2.4)** 2. the content should be deeper, but not board without any deep investigation, **(2.3)** more algorithm part. **(2.2)**

less work load! less lectures or labs! It is not possible to follow the lectures because of the stress the labs create **(1.3)**

I think there are MANY improvements; It is literally IMPOSSIBLE to understand the professor X. I think it does not make sense that a person who does not articulate the words in English is teaching in an English course. This made me very angry since his lessons were a loss of time, I could not understand anything he explained. It happened the same to the whole class, and seems that this was also the case last year with previous students who also complained about his speech; **(2.4)** The course is called Introduction to Bioinformatics. I do not understand why we get to the computer labs and we are supposed to do, without any prior explanation from the TAs, programming exercises that require some level of knowledge. **(5.1)** Then, if you ask the TAs for help, they barely help you with the exercises. The course was overall very frustrating and disappointing; **(5.2)** The other lecturer, Y, seems like he did not prepare his slides at all. He stared at them for a long time before knowing what it all was about. Also, it was impossible to get information from his slides, as he just copied the pictures from a book that we had to read if we wanted to get to understand anything about the topic. **(2.4)**

Mainly pedagogical improvements are required. The main lecturer X in particular should make a greater attempt at engaging students and making his lectures more understandable. **(2.4)**

1. The course would benefit from being organized in a such a way, that we would not have labs/assignments on a subject before having a lecture covering it. This does make us spend a lot of time learning about the subject in a sub-optimal and time-inefficient way. The hours wasted on this sadly do matter in this course and are going to be missed dearly; 2. Lab sessions shifts and timing of lectures. Being in the afternoon shift easily puts one behind schedule. An interesting statistical experiment could be to see if final exam score correlates with shift assignment. While planning and organisational skills are important to develop, in reality a lot of productive hours somehow disappear when this schedule is imposed. The timing of lectures also coincides with insulin coma after lunch. I would suggest early morning lecture and one shift laboratory session for all if more computer halls can be booked; **(3.2)** 3. The course could benefit from diversifying the assignments: instead of written assignment for all topics, one can be grilled in form of oral exam, whiteboard coding or article presentation **(4.1)**. I am sure you know that in the current state of the course, both workload and pace are very high. This might feel great at first, but in the last two weeks, there was no time to REALLY internalize, consider and reflect on the rather difficult underlying concepts. All of the time (in the day and most of weekends) goes towards many things required to be on time for this course, that there is no time remaining to really care about the subjects. There is really no time to open any curiosity doors and be motivated to learn more and more. **(1.3)** For most of the things we have to be satisfied with some half baked hasty interpretations before moving on to something else. This might be counter-intuitive, but at some point the retention really suffers when there is no time to ask questions and be curious anymore. **(2.3)**

First the way of teaching. This is a INTRODUCTION to bioinformatics. I still remember the first day of class, most of us has NEVER done anything in programming, and they (the TAs) just gave us a link to a tutorial and let us work on it. Not even an explanation or introductory presentation. I could have done exactly the same at home. And it followed like that in the whole course: the TAs do not help you at all, just tell you to google things and figure out by yourself how to do everything. Honestly, I could have done exactly the same at home, since their help is INEXISTENT. Plus the university can save money instead of spending it on teacher assistants that do not teach you anything. Also, the TAs were joking about our null knowledge on maths or physics. They do not even know our background, and were undervaluing everytime that they could. That, in my opinion, is a severe disrespect for the students and should never be tolerated. **(5.2)** - A course taught in English for an international audience should have a professor that speaks an understandable English. X is not able to teach in English, so I highly recommend to hire another professor or a translator, cause otherwise is not possible to follow the class. I had to learn everything from other lectures /papers /additional material that I found on the internet. - Y does not prepare the classes at all in my opinion. He just tells us to read a chapter in advance from a book, and then he just puts photos from the same book and explain random things (he even skip slides cause he does not have anything to say from the picture, sometimes it seems like he did not even do the ppt himself). Again, all I learnt was directly from the book, which is something that I could have done from home). **(2.4)**

The amount of information exceeds the humanly possible ability to obtain that information in the given timeframe. The information itself is good and important, but there is no time to understand it. I would suggest to either span the course more evenly in a longer timeframe **(1.4)** or design it in separate courses: one for the computer lab and one for the lectures, **(3.2)** however, the previous option will only be beneficial if it is also managed in longer time. **(1.4)** If extra time is not an option, lower the amount of labwork, since this takes the bulk of the course time and is not even covered nor gives any extra points in the actual evaluation of the course. **(5.1)** If this is also not an option, have a student-friendlier approach in helping students with labwork so the amount in the lab is spent more constructively contrasting to the amount of time students have to google instead of getting help from the teachers. **(5.2)** I would also suggest incorporating labwork to course evaluation. If not for determining the grade, then providing additional points to students who have managed to complete the lab as intended. **(4.3)**

It was a very intense course and the programing parts can be worth to reschedule as an own block in the course **(3.2)**

The course is very packed. The computer labs to learn a new (programming) language and a lecture with a new topic almost every day is too much. I just was only able to concentrate on computer labs and the assignments there and had almost no time to prepare or refinish the lectures. **(1.3)** In addition, for someone who never programmed before 3h for one assignment is not enough and if you need extra time for the programming part you have less for lecture preparation. **(1.3)** Especially the lectures given by X were really difficult to follow and to understand. I also got the impression that Prof. X does not really care about the actually knowledge transfer and that the students understand and follow him. He also does not correct mistakes on his slides students point out in the lecture. As an additional example that can be seen in the videos he uploaded on youtube, which are very sloppy for many parts ("explaining" 5 slides in 30 sec) and he is not clearly speaking (mumbling). To be honest, I am really disappointed that a Professor at SU is giving that bad lectures. **(2.4)**

It requires high level hearing to understand the teacher… **(2.4)**

Better lectures at slower pace. **(2.4)** Slides with more information. **(3.3)** Less content in the curriculum or provide more time. **(1.2)** Less pressure and stress on students. Both lab work and lectures together are very difficult to handle in such a short amount of time. **(1.3)**

Less lab work so that we can have more time for the lecture **(3.2)**

-Force the professors, teachers and TAs to read, apply and respect the course syllabus and the rules here on Stockholm University! The things that are not stated in the course syllabus are considered to be illegal! **(3.1)** -Decrease the number of labs! **(1.3)** -Force the TAs to take a social skill class. **(5.2)** - DO NOT have this preposterous rule that if one does not finish labs one week after the labs was done, one can only obtain an E as a final grade, independent of how well on did on the exam! Especially when it was stated in the beginning of the course that the labs are not to be part of the grading! Furthermore, it says NOWHERE in the course syllabus that this is how the course is to be graded! If you have any interest in designing a course that is desirable and useful, take away this ludacris rule and start being more reasonable! **(4.3)** You will win no favors from the students by stressing them with lab work and punishing them if they do not submit and pass a lab after a week, especially when we av 3-4 labs a week!!!! The only thing you will end up with are stressed students that will curse the name of the course and spread the word that this course is horrendous! And as this course is mandatory for the masters program in biochemistry, students will not apply to the program. It is not like there are students flocking to the program as it is! **(1.3)**

longer period of time or/and fewer lecture topics **(1.2)**

Give me more time to solve the assignments and reduce the number of deadlines **(1.3)**

a) CUT DOWN the INCREDIBLY STRESSFUL amount OF WORK! It's completely unrealistic to expect people do this much in such a short time and not be freaked out about it. The 3 hour timeslot is absolutely not enough for most of the sessions and I ended up attending both morning and afternoon sessions and then working also home. It's insane! I hated every minute of this because of the amount of work!

Also, by answering mechanically so many questions, I actually don't remember what I did at the beginning! Less is sometimes more! Also, I could not care less about the lectures, since I had 3-5 deadlines ticking behind my back every week. Suggested improvement: Either cut some of the assignments or reduce the amount of tasks in the assignments by 1/3. Another option is to make the course a full 15 credit course and then the amount of assignments is reasonable. **(1.3)** b) REMOVE the insane 1 week deadline rule or you get an E! Several problems with this: -I don't think the rules of the university for allowed grading are followed here and it's a breach of my right as a student to be graded fairly, if you guys just make up a rule! I'm not sure yet, but I might be taking this up somehere and complain about this. YOU ARE NOT ALLOWED TO MAKE UP RANDOM RULES! -It's incredibly unfair? I mean, not only was I stressed out about the amount of learning, the fact that I didn't have time to learn for my exam, but a week after the exam I was not sure whether I would be eligible for a higher mark than an E, since I received no response for few days on my last lab report. It SUCKS to hand in 15 assignments, but get downgraded AUTOMATICALLY to an E after all the hard work I've done!!! It's very demotivating and stressful! Suggested improvement: If you must have the rule (and I can see why some form of it is necessary), make it scale, for god's sake. For example after 1-2 missed deadlines, you will be missing points enough to get an A and then after more missed deadlines B, C etc. But not DIRECTLY and AUTOMATICALLY to E, this is insane! Also, explain it in the course syllabus. I'm still sure this wasn't a "legal" rule to introduce in this form. Follow the rules for making the rules! **(4.3)** c) Please instruct the TA's to not talk about their political opinions in the computer room? I mean I don't think this was a huge issue and that some dramatic things were said, but I left the room on several occasions (to not get into an argument), ie. when a TA was discussing how trans people actually think they're one gender, but they're not - look, it's as if I was listening to my racist uncle at a family dinner. I see no reason why I need to listen to this stuff in a Bioinformatics course. I don't think this was a huge issue and everybody is entitled to their own opinion, but please let's try to stick to teaching bioinformatics and not randomly bring stupid stuff like this up for no reason. Suggested improvement: Just mention this to TA's. It's not that I'm offended, but that I would actually stand up and get into an argument with them about something completely irrelevant to the subject. **(5.2)** d) Please try and change the schedule, in the sense that we had the exam on Wednesday and our next subject began on Friday - why not have the exam on Friday and begin the next course next Monday? Suggested solution: If possible, try to negotiate a more logical schedule with whoever is in charge of that. **(3.2)** e) The "plagiarism" accusations. A couple of problems with this. -Most of the sources listed are wikipedia links. Many of the questions are literally asking us to go to Wikipedia and rewrite what is said there. **(3.3)** When we ask TA's, they tell us to find out on the internet. **(5.2)** Result? The way the assignments are designed is that they're systematically pushing us towards being dangerously close to plagiarism accusations, because we're asked to plagiarise stuff and then to cover it up by rewriting the sentences!!! Suggested solution: Reduce the number of questions that ask for this, rephrase the questions, include better sources than wikipedia, **(3.1)** instruct the TA's so that "find out on Google, I'm not telling you" is not their default answer. -We've literally been said that we should go online and copy programming code and try it out (since it's a computer code) and that the only consequence of that would be that we would have to be able to explain the mechanics of how it works. That was a complete citation from one of the TA's **(5.2)** When we follow this advice, some of us get sent directly to the board for plagiarism. That's such an incredibly outrageous way to go about this! ALSO, I believe plagiarism accusations should be raised AFTER a consultation with the student OR when there's a REASONABLE CASE for assuming that it was the student's intent to circumvent the learning process and better themselves. Especially since we've been basically following the instructions!! (As a sidenote, I personally wasn't accused of plagiarism, but some of my friends were and I'm incredibly outraged by how the people involved in this subject handled this). Suggested solutions: Rely less on URKUND and actually think before raising plagiarism accusations. Especially if you give FALSE INSTRUCTIONS to GO OUT AND COPY STUFF and then hypocritically act as if that didn't happen!!! Like - you instruct and nudge people towards plagiaristic behaviour and then you accuse them of malicious intent (!?). **(3.1)**

Some of the lectures seemed overpacked with information. **(1.2)** Instead of presenting multiple versions of algorithms the focus should have been on the conceptual understanding of the necessity for the algorithm etc. **(2.2)**

**Course Evaluation 2019**

Nil

As mentioned above the course content was great. However, the workload was not really balanced and a bit unrealistic. I would say that I could manage the course, but not with also keeping my physical and mental health intact. My suggestions would be to decrease the number of mandatory events. I understand the concept of having mandatory quizzes and readings, however, it adds on so much stress and you do not even have time to be sick one day without getting so behind that you get the feeling that it is not even worth to continue the course. It is important to include time for rest and to take in everything you learn also. Maybe there is a way of decreasing the number of examined/mandatory events because to be examined every day is a bit too much. **(1.3)**

I did not like the test. The multiple choice questions were sometimes a bit arbitrary and did not really test general understanding, more random facts learned by heart. **(4.2)** Short sentence answer questions are more work to correct but might be better to access knowledge. (Maybe a task where we code, or do something on the softwares online) **(4.1)** Maybe it would be good to send the course reads in advance (in the first semester we would have had time to invest in that, which would have been good in the course) **(3.1)**

It would be better if there could be more introduction about the software or program that will be used before each lab. **(5.2)**

Simplify the contents of lectures. **(2.2)**

Disminish the amount of readings per lecture. I think is better for us to read less pages but be able to understand everything that we read. If we have too many things to read we end up skipping those parts that we don’t understand, which reduces what we learn. **(1.1)**

1. The course was too big and it was fit in a month while it should be at least 2. The ECTS credits form the hours spent in this course were not even the half that they should be. The workload was too much and we were expected to work on the course 10-12 hours per day something that I think is unrealistic since a human brain cannot absorb all this information. **(1.4)** 2. I am not sure about the focus of the course. After having completed it I think that I learned structural biology and not bioinformatics. I understand that structural biology is important to understand the bioinformatics part but at the same time we were not told which tools should be used in each case and how to do it properly. **(2.2)** Even the exam questions were memorizing, strucures, aminoacids, and peptide bonds and not how to use it in bioinformatics. The exam should be a real case bioinformatics problem that we should describe which steps and which aspects we would take into accound to solve it and not memorizing the slides that we were tought during the lectures. **(4.2)** 3. The labs were not well organized. Some were extremely short and were only based in theory while others were more than they should. In a three hour lab we were supposed to run I code twice that each time took 2 hours in the best case. Furthermore, the bioinformatics aspect was not clear in all labs. **(5.1)** 4. Every day we were supposed to read before going to the lecture and do a pre lecture quiz. In principle this idea is really good but it was working only at the first 3-4 lectures where the readings were not really big and heavy and could be done the afternoon after 2 hours of lecture and 3 hours of lab. **(1.1)** Furthermore at the beggining the lab quizes could be answered from the assigned reading, while at the second half of the course the reading contained absolutely none information of the ones needed to answer the pre lecture quiz and we had to search extra information online, making the reading useless. **(4.2)** Furthermore, we were assigned a reading of 200 pages in a scheduled time of 2 hours which is imposible. This was clear since the lecture reffered to this particular reading could not be completed in 2 hours but in 4. **(1.1)** Furhtermore, we were assigned readings from books that we didn´t have access to which I think is unacceptable. **(3.3)** Finally, I am not sure if the people giving the lecture had any idea about the contect of the reading assigned because many times we spent time reading something that was unrelated with the pre lecture quiz and the lecture itself. **(4.2)** 5. In the schedule it was not taken into account that after the pre lecture reading and the lecture we needed to spent AT LEAST 3 extra hours to put in order everything that we learned. This was not scheduled and the weekends were not enough. This brings me back to the overload of work that made our lives really really hard this bioinformatics month. 6. The secret sequence assignment COULD NOT be completed within the 2 days scheduled and there was not any time for that in the schedule. **(1.4)** 7. After the first partial exam and intense study days (more than 12 hours per day) we had to read 100 pages to do the pre lecture quiz making us even more exhausted and stressed. **(1.1)**

More emphasis on application, such as more or longer labs if possible. These are really useful and the tools and resources used in the labs are what you would actually use in your work. **(5.1)** Less emphasis on the exams, in terms of their total percentage contribution to the course, giving more time to labs and more credit to labs (since we spent three hours a day on them). Overall, more emphasis on labs/projects and less emphasis on the exams. I felt I learned the most with the labs and had the most fun with the project, but would have liked to spend more time on the project. **(4.3)** Having the project due right after the exam was very stressful and I did not get to do as much as I wanted to in the project. **(3.2)** Also, having two exams was very tiring and stressful with how much is already happening in the course **(1.2)**. Perhaps if there are two exams, have more broad essay questions, since this course covers a lot of material, **(4.1)** and have the exams worth less. **(4.3)** The exams had very specific questions and it was quite frustrating when you spent hours studying and reviewing all of the material to get tested on a few, very specific details. **(4.2)** Even if the exams are online and even if there is only one, have more essay questions that are broad and make you think critically, rather than the more memorization based exams these past ones were. **(4.1)** Some of the later labs that used MMB were really difficult to finish in 3 hours, so have those labs span more days so we can let MMB run overnight. **(5.1)** In the labs themselves, have less theory/review questions on the lab quizzes and more questions actually using the tools. If you want more theory/review questions, you could have post-lecture quizzes in addition to pre-lecture quizzes (4.2) that are separate from the lab quizzes. This way the lab quizzes really focus more on using the bioinformatics tools and have us do even more with these tools during the labs **(5.1)** Also, do not assign more than 50 pages of reading in an evening. It can take a hour to read 10 pages so more than 50 pages is too much for one night. **(1.1)**

I think the content and time did not match. We may extend this course or we can deduct some lab **(1.3)** or content which are not relevant. **(1.2)** In the other hand, I appreciated with every content.

There are several aspects of the course that needs improvement. For example, the reading requirements were often a bit much, so try to reduce this by providing specific pages to focus on (as once provided when we originally were supposed to read 100 pages in one day). **(1.1)** In general, the course is very intense and since this topic is new for many students, perhaps it's not reasonable to give this course with this amount of content in one month **(1.2)**. Even though you pass the exam and all quizzes and labs, the hectic environment takes away the fun from the course (which is sad since the content is very interesting) and a slightly slower tempo would most likely be associated with greater learning outcomes **(1.3)**. It would also have been nice to spend more time on the secret sequence report which felt slightly rushed since we had to prepare things like labs, reading before lectures and pre-lecture quizzes during the whole course, which gave limited space to only focus on the report. **(1.4)**

Better Organisation of the additional material, smaller pdf sizes! (they took up far too much storage space without reason),**(3.3)** add more programming (but not on top!) **(5.3)** less pages for the reading assignments **(1.1)**

the needed reading what should be prepared before every lecture was sometimes too much and too many pages, **(1.1)** while it was not really said which aspcts are important on that sides --> moreover it was not really clear with aspects were important for the exam and some very complicated aspects were not important in the end to know; some aspects, escpailly informatic or deep mathmatic aspects, were hard to understand for a student with biochemical background --> better: more illustrations; and the two exams were not too easy in my opinion!!! it was possible to pass them with a good grade, if everything was learnt and understood! **(2.4)**

A little more time should be given for the course. **(1.4)**

The lessons are okey but the exams should be more accurate with them **(4.2)** .During the lab I constantly felt that I was expected to know programming, I would recommend requiring computer skills for future students in order to take the course, **(5.3)** or if you want to still keep it accessible to everybody give lectures about this before the labs (consider there might be people that do not even know what is the terminal). I felt it is a course planned for programers to learn about biology and not vice versa. **(2.2)**

Try to make it less demanding in terms of time. We are enrolled full time but that should not mean that you have to dedicate 100% of your day only to the course. **(1.4)**

Less theory. Better teachers. **(2.4)** The labs are too messy. **(5.1)** Follow less the book. **(3.2)** Less content but more clear. **(1.2)**

The part of the course before exam was very clear as it followed a good flow like a story which helped us visualise how one would go through the different softwares and analysis for any random given sequence. However, the second part of the course that was based in structure prediction was more complex to understand the clear flow **(2.4)**. Even though the concepts were clear it was a bit difficult to say how to apply any of it when solving an actual problem of structure prediction. **(5.1)**

highlight the focus of preview content, instead of the whole chapter **(1.1)**

The loadwork. The reading materials are impossible as we also need to finish 2 quizzes everyday **(1.1)**. There should be group works. I usually learn more from group work as we can share ideas. But, in this course we usually do everything individually. **(3.2)**

Time distribution for the preparation of the class(reading task).**(1.1)** More organized teaching method during the lab. **(5.2)**

This is definitely not a one month course. The workload was always too much. The stress during the whole month was too high all the time. The same content should be taught but in a two months period. **(1.4)** It is not feasible to study 100 page for the next day after having 5 hours of lectures and labs. **(1.1)**

Lectures **(2.4)**

**Course Evaluation 2020**

It should be a 15 credit course, not enough time to fully absorb all the information, especially for the students with no computer science background **(1.4)**

Perhaps distribute the chapters in a more equal number between partial exams. I felt the first partial exam had much more topics to study comparing to the last one. **(4.2)** The chapters taught by Professor X were somehow very blurry and difficult to understand during classes. **(2.4)**

Some labs were definitely too long or difficult. They should be shortened/simplified. There were also two labs that were definitely too short/too easy **(5.1)**.

The preparation for the lectures (i.e. pre-lecture quizzes) was way too time-consuming. Even after finishing with all the assigned reading, I sometimes could not answer the questions without additional reading. **(4.2)** I spent more time on the quizzes than on the exam preparation which does not match with the respective impact on the final grade. **(4.3)** It got better in the second part of the course though! Also, it would be great if ALL lecturers could use their slides in class to illustrate the topic! **(2.4)**

Less learning material so students that are not so experienced have time to process it and have enough time for all the quizzes. **(1.2)**

Alternate the labs so one do not have to attend the same time slot for 5 weeks (by switching the lab schedule after the first exam). **(3.2)** Reduce the amount of reading material. As for now, it was all about quantity instead of quality and there was no time to go more into depth regarding the various topics that where covered. **(1.1)** Have more time for programming introduction. **(5.3)**

- spend more time with actual code writing in the labs; - In the labs it would be helpful to discuss the code of the labquiz together to get a deeper understanding of whats happening in the script; **(5.3)** - reduce the workload! Most important improvement! The topics of the course are very interesting and I see the difficulty to cover all the information in such a short period of time. **(1.2)** But because of all the reading, there was no time to understand the topics deeply or just go back to code from the lab and try to solve the problem in another way out of interest. (the labs were designed in a good way but the pre-lecture reading

and quiz take to much time); **(1.1)** - The book "PROTEIN STRUCTURE," Understanding Bioinformatics, Zvelebilt & Baum. is quite unintuitive to read, I would prefer different reading material. The first two books are very good to read and well structured! **(3.3)** - In the second part of the course, the lectures were very good to follow! But in the first part of the course, the lectures until the first exam were too fast and unstructured in my opinion. Maybe use the first lectures to shortly sum up the topic of the reading for that day and then go into more detail on certain topics which need more focus. **(2.4)**

Workload and time management. It was kind of impossible with the availability issues of the course material to prepare all suggested readings before the lectures. Some quizzes also took me way over the calculated four hours which ended up with me studying over 8 hours some days without even starting the reading. Doing anything besides university meant that you had to cut down on the readings and therefore lack information you need for the exam. **(1.1)** And please check for false/ not very clear questions in the exam, **(4.2)** I personally prefer having the exam questions on paper at least as I like to work with the text and cannot extract information from digital media that easily. **(4.1)** The grading is much based on knowledge and not on applications, I know the labs and pre-lecture quizzes are meant to improve that, but the theory of the exams is so far from the application from the lab, that it is difficult to connect those. For the first partial exam it would have been nice to know beforehand that the focus will not be biological but more math related and to make the students clear that if they cannot do the math calculation, logarithms etc. in there head, that they should maybe look at it again, as it might be required in the exam. **(4.2)** For the Labs, it would be really nice if for the labs all TAs could have an overview what is gonna be asked so we do not need to wait for the one making the quizz to ask specific questions on its content. **(5.2)**

too many things in very short period **(1.4)**

With all respect, the teachers that gave the lectures NEED to do a teaching course. They do not know how to comunicate. And over all, it is obvious that Y does not prepare the lectures as things that we suppose to know for the pre-quizzes he was not able to explain in the lecture. **(2.4)**

Better specify what to focus in the pre lecture readings, sometimes we had to read lots of pages that were not even mentioned during the lectures. In this cases I stopped reading at some point because it was too much information but then realized I had missed some important things and invested a lot of time on contents that were not that relevant for the course. **(1.1)**

Allow more time for lab completion. The computer labs were very interesting, fun and where most of the active learning was done. I think it would be more beneficial to allow more time for lab completion, rather than omitting the lab content. The extra python exercises were always very useful and fun as well. **(1.4)**

Very high workload. Please start slower at the first day(s), to give students who start at su in the summer semester a chance to go to some welcome activities. **(1.4)**

Some of the Labs were too difficult for us, especially for those who do not have the background in computer programming. The machine learning lab was too tough. **(5.1)** I think you should at least increase the deadline of lab quiz to next day instead of on the day of lab **(1.4)**

Change the time schedule, giving some break between the lecture and lab. **(3.2)** For the lecture, I can not understand what teachers are saying sometimes, because they speak too fast and unclear, and maybe too many catchphrases. That is not good for non-native English user. And why sometimes the teacher seems like also don't know the right answer… And for the record, the angle and view for the camera are bad sometimes. The teacher was calculated and wrote on the side whiteboard which was not concluded in the view of the camera… For recording the slides... it actually doesn't have many notes to see. The teacher's drawing software always have problems. Maybe try to use Notability or other Apps to improve the lecture writing. **(2.4)**

lecture preparation (examples demonstrated in lecture). **(2)** allow comments for each question in exam, as was the case at the second exam. **(4.1)**

Make it a period A-B course **(1.4)** and expand on the practical components **(5.1)**

Some of the labs could have been slightly shorter as they were quite time intensive. Towards the end, some of the labs had a few theory based learning questions that could have been included in the pre-lecture quizzes so that some time is saved in the lab quiz. This would be nice as generally most days were from 09.00 - 18.30, and no real lunch break because the lecture and lab was back to back. **(1.4)**

a little bit intensive **(1)**

Workload is quite high per week so it may be adjusted a little bit. **(1)**

More time to study in depth **(2.3)** than just a large syllabus and short time **(1.4)**

During the labs, many exercises were clicking through websites. For sure this is necessary to get to know different websites which are necessary for secondary structure prediction for example but it would be nice to include a bit more programming. **(5.3)** Regarding the lectures: It was really a lot to read **(1.1)** and less topics **(1.2)** / 'deeper' discussion of the individual topics would be good **(2.3)**. Instead of covering so many topics I would have liked to discuss less topics **(1.2)** but those more in detail **(2.3)**. (Thank you for reducing the workload when we asked for it because we really struggled to handle the reading.)

Less to no lectures from X and definitely **(2.4)** no exam questions from him. **(4.2)** A little more preparation from Y for his lectures, especially when it comes to complex concepts. **(2.4)**

Better organization, especially in the beginning, regarding schedules, canvas platform etc **(3.1)** Less volume of theory, **(1.2)** more time to work on the labs **(1.4)**

I recommend that the professors prepare for the lectures, as in some cases they were unable to solve key questions regarding the lecture (pairwise alignment, XOR gate example). Moreover, X's lectures were especially unorganized, and very difficult to follow in the absence of slides. **(2.4)** Most importantly, the first exam did not cover the most relevant aspects of the course content, in my opinion. There were few questions, manly focused on specific details (complexity, very specific formulas never introduced during the lectures, machine learning) while broader questions about phylogenetic methods or alignment strategies were not included. As I see it, the exam did not reflect at all the fact that a student has studied/learned the content. **(4.2)**

I often percieved the lectures as quite unstructured. I also had a really hard time following the lectures, much more so than in other courses. And I often got so lost that it made me feel stupid, so I really didn't dare to ask questions. Especially because when some people were following I felt like it might be just me. **(2.4)**

I don't think of any improvement needed for this course. maybe improve the secret sequence assignment. **(4)**

Please remove annoying quizzes. In my opinion, they were not of any help, they just created lot of stress and stole my time which i wanted to dedicate for reading lecture topics from books. Even when I didn't get sufficient time to read books, I tried reading anyway. It got me low points but thats ok. I want to understand the basics and build on it.Please encourage book reading, ''exams from slides culture'' needs to end. **(1.3)** Anyone can just read slides and get good points in exams. By the way whats the point of pre lecture quiz and being graded for that? I mean if I go to lecture, it means I donot have sufficient knowledge and I want to learn about certain topic. If I already read the topic (since one needs good

points in pre lecture quiz), whats the point of lectures? Not all are A grade students. I would also like to request Y to review the grading criteria. Anyone can cheat on quizzes and exams to get higher points. During the partial exams, the seating arrangement was not good. Since exams were computer based and had mostly multiple choice questions, I think it was easy to cheat from other students. One can just sit behind some good students or near his/her buddy and get good points. The assistants were just sitting together and were busy in their stuff. They were not watching all the time. I think my laptop screen was very visible to the students seating behind me. There should be a lot more distance between students in the examination room. Also the students going to the restrooms should be checked for cheating material. I have moral high ground so I didnot do any cheating but it doesnot matter. In the end, its all about

points which I am going to get less for sure. I think secret sequence report and two partial exams are enough. Not everything needs to be graded. I have more suggestion. **(4.3)** The emphasis should be given in making biologists, cell biologists, molecular biologists, biohchemists etc skilled in bioinformatics so that they can use those techniques in the project work or thesis. Some topics were covered in too much detail eg machine learning. I fail to understand when and where non bioinformatics master students are going to need that?? I think more stress should be given on use of various databases, BLAST, modelling, conserved residues studies, multiple sequence alignment and other similar tools. Its better to

be master of basics than master of none. **(2.2)**

Lectures should be improved. It is not the content but the way it is explained. You cannot expect that we read 30 pages per day and the teacher just comes and reads the slides without really knowing how to explain things when asked. I think we know Y had the best of intentions, but did not prepare the lecture (that is why lectures more related to structure were more fluid and could answer without a problem). X is simply horrible, random stuff, disorganized and he even gave a 15-minute lecture unrelated to the slides, and the day of questions for the exam he actually gave the lecture with the slides.

It may be impossible, but I think that some of the assistants for the labs could take some of the lectures. I don't mean all, but phylogeny could be a good idea, since the class was not clear at all and phylogeny is so interesting and useful when understood. **(2.4)**

**Course Evaluation 2021**

Lecture style **(2)**

dedicate more time to unix lab link contents from computer labs better, so that students really see how things work together **(5.3)**

Extend the time for the labs in the first week **(1.4)** a few more words for the secret sequence **(4.1)**

I wish we were actually thought the programming part of this course. I was before the course very excited to learn bash and python. But it was very clear to me that the teaching and the exercises was not made for beginners. So i would not like to cut it out for the beginners like some students mentioned in the course. Instead give us an actual python lecture/lectures before we have the python assignments. I think the bash commands was fine to learn on my own, but python needs more teaching.**(5.3)** The PDF which explained the basics of python was super helpful. But there was no time to go trough it properly before we were thrown into the 4 python scripts on the second day of the course, which then took me and some other beginners until midnight. **(1.4)** For those exercises I feel like I would learn much more if we were divided into groups with beginners and non beginners, where the beginner group can go trough the exercises together. The dividing of the breakout rooms worked great for the other parts of the course but not this specifically, since the level of knowledge was so divided. Then having to show your screen on zoom to the rest of the group when you are still on the first step of the first exercise after 3 hours felt daunting. **(3.2)**

In the theory, sometimes it feels like no point is reached after 30 min of explanation. Maybe more concise what is what we are trying to learn. **(2.4)**

Y lectures were a bit harder to follow. It would be nice if he could be more clear/straightforward with his explanations, both during lectures and discussions **(2.4)**

Make the labs so that you understand better how we would use some of the tools in real life. **(5)** I found that in the secret sequence project that I knew what tools to use and what they did but was not shure what could be interesting to use them for in reaserch. **(2.3)**

It would be awesome to have more time during the course to be less stressed: increasing the number of weeks for this course it's necessary in my opinion **(1.4)**

Give us the lecture to watch before the first class earlier than the weekend before class **(3.1)**

First and foremost, having proper notice of what to expect at least a week from the start of the course rather than the Friday evening before the break weekend before our first and second semester (MTLS), especially considering that we already had homework to do for Monday (not super reasonable). **(3.1)** Making sure labs are more collaborative (this was highly dependent on lab group and TA per group) **(5.1)** and on top of that, that students are all treated equally. What I mean by this last point is that I was placed in a group that was mostly male-dominated and many times I was the only girl attending/participating, which doesn't feel super comfortable considering that the dynamic is overall dismissive/interruptive of my ideas by nature. I'm not being overly sensitive, I have thought this feedback over and over, at the risk of losing anonymity, so I am simply trying to raise a genuine concern. I'm not saying I didn't appreciate my male colleagues' contributions or insights, but I am saying that I would have felt WAY more comfortable sharing mine if the power dynamics weren't so off-balance. I understand that the groups were randomly generated but ensuring a female-to-male ratio that's closer to 50/50 in every group would have benefited my overall learning experience during the group work, on top of the fact that a more diverse group would generate better/more interesting discussions rather than one-sided ones. I hope you take my feedback into consideration for future lab group assortment. Thank you. **(3.2)**

The first days were pretty bussy, I would try to spread the workload a bit more over the course duration time. **(1.4)**

Use computer-written slides and make the live lectures and labs from 16.00 onwards to allow everyone to participate. **(3.2)**

Extending the deadline for the lab quizzes especially for the first week as I found the terminal exercises quite intense. **(1.4)** Provide the slides always! I'm a big fan of writing over them to complete the information. Find other (better) supplementary material. it seemed the book wasn't updated and contained some errors. **(3.3)**

The secret sequence report had either too little word limit or too much mandatory questions to properly explore and expand the research. **(4.1)**

An amateur person needs little bit of extra or more time to understand the concepts of Python programming in this Bioinformatic field. **(1.4)**

Teaching the linux environment and python is a great thing if done right. As the available time is limited, the way of teaching should be fitting for that. As an example. Listening the lecture/reading is great for information uptake. The quizzes and the inverted classrooms make you think about it. Also very good. The labs on the other hand are partly good and have some downsides. When it comes to Python and Linux, the environment and the programming language is completely new to some students. The problem from my perspective is, that these students have trouble moving around in this new Environment and being in trouble costs time. That we dont have. This means 3 things. 1. Getting started: Everything we see is new and we feel uncomfortable and clueless about what to do. The introduction has to pick us up just at this point. It might be difficult for advanced people to think about what they needed to know when they started, so here are some ideas: A map of the server folder structure for orientation would be very helpful. More Graphics in the Bash Introduction script by TA, which is nice to read, but needs more graphics to explain what the commands do in a visual way. For example display ./ ../ pwd cd etc. on the map. A graphic about the way we work with the linux terminal on the server would be nice. I had no clear idea where and how my programm was accessing the bioinfo server.

These graphics help us to connect our thinking of visual folders and functions to a text-based command-line-level! The colourful BASH environment is definitely a gamechanger! As the colours clear things up a bit. Put it in the script please. A graphic about what and why we are doing it when for example doing a BLAST search in the terminal would be great for orientation. I learned the most there, when I looked at the files step by step that you had given us, like the small Database and the file structure of the fasta file. This knowledge helped me the most in understanding what I was doing there. Maybe give us task to understand the file strucutre better, as this helps very much in understanding. 2. Teaching commands: Please please please: If you show/tell us something in the script: Explain it directly and give us a use for it, so we can learn while doing something. If you show us 100 commands in Linux or functions in Python (especially in Python) first and then tell us to do something with it, it is EXTREMELY TIME CONSUMING TO FIND WHAT I NEED FOR WHAT I AM DOING!!! Some may argue that it is great for the learning outcome when the students have to think about the tools, and I would partly agree, but especially for Python, the level of complexity of what we can do with the tools increases exponentially. It is like giving a kid a dictionary and expecting it to speak. Please do it like this: Show us a command (or a very LIMITED number of commands), tell us and show us graphically what it does (showing the expected command lines is great, as TA has done it), and then make us use the tool by giving us a small task. This way we learn the tools fast and can then later on use them for more complex tasks. You should first give us a hammer and show us how to nail a nail and then (!) give us a more complex task like building a wooded shed. 3: The learning outcome of the python parts: What was the goal? If the goal of the python labs was to help us understand the lecture topics in terms of what the computer does, you have overdone it completely. What you did here was a Python course that could have been a seperate course and not a few afternoon lab sessions. I have talked to several people that knew Python and asked them for help, and they all commented on the workload: "You have to learn the Theory behind Python and all these commands in an afternoon and then apply them for solving these tasks ? It took me 4 weeks to be able

to do that." They helped me with some files and even with their help it took me the whole day to get them done. I would urge you to really think about the intended learning goal with Python there and limit the learned tools to that. I hope I could provide you with some input to make this course even better :)

Thanks for reading this. **(5.3)**

More lessons on genomics/transcriptomics, even as a trade of with some structural informatics. **(2.2)** Maybe sometimes try to connect 2-3 labs that tasks relate to each other between the topics, if possible. **(3.2)**

1.I would announce the material and the schedule for the course way earlier. Not only a weekend before as it happened this year. Especially that we had to come prepared for Monday classes. Definitely announced too late **(3.1)** 2.The discussions could be more structured in a way that there is time for students' questions as well as some brain-storming about relevant topics that the teacher and TAs find as relevant. Therefore, instead of suggesting "discuss what you found most interesting/difficult" I would prefer a list of more specific topics that should be discussed within each group. **(2.4)** 3.Would be great to have an access to the slides from the videos provided with some notes to each slide that highlight the most relevant objectives of the lecture. **(3.3)** Sometimes the videos had a poor quality sound and it was hard to understand the lecturer. **(2.1)**

As the course is supposed to welcome student without any knowledge in coding, it would be a good idea to start with the basics or to make two groups according to their level in coding. **(5.3)** The lectures were not very clear sometimes and even confusing at some points. **(2.4)**

I think there should be a little more material dedicated to some knowledge of neural nets and machine learning as these concepts were tested but i dont feel they were covered very well. **(2.3)**

It would be nice if we could choose a sequence We are actually interested in for the project. Also it would be extra nice if it was in pairs/small groups. **(4.1)** The workload of this course is HUGE and Highly individual so it can be better if We can Exchange some knowledge and entourage team Work. **(1)**

The scheduled time seemed a bit short sometimes for the labs, which got us very exhausted, which was bad because the next days recorded lectures was hard to understand after hours of focusing on coding for example. **(1.4)** Other improvement would be regarding the evaluation of the final report, as I we still got no result after two weeks. **(4.3)**

The first week was very intense, probably because we got the information about the course just before its start. **(3.1)**

**Course Evaluation 2022**

some of the lectures were hard to follow due to speed and accent of the teacher **(2.4)**

In my opinion X‘s pre-recorded lectures were really hard to understand (acoustically). It was hard to connect information or see the connection. He should really consider to re-recorded is lectures (see Y‘s lectures with structure) and enable subtitles for them on youtube. **(2.1)** I think the discussion were not really well structured. Discussing in breakout sessions what we found most interesting etc. is not pretty helpful (at least to me). They should be more interactive in terms of engaging discussion in the main session about difficult topics (maybe check last years exams and see what questions student‘s struggled most with. **(2.4)** The pre-exam sessions didin’t really have an inpact on me. **(3.2)** Maybe the lectures could prepare topics that we should focus on and shortly go through them with us. **(2.3)** I didin’t like exam.net at all. In the begining of the course it was said that we would have access to all media we might need. I adjusted my preparation to that. **(4.1)**

The course video of the second part of the course should be improved. There should be subtitles, sometimes the narrator speaks too fast, and the videos should be collected into one video, for better course structure. **(2.1)**

I would suggest the lecture content to be more focused and more clear on specific learning outcomes. There are many things good to know, and it is definitely important to have a good understanding of how the tool we use work. However, it is sometimes very unclear what information is more important to carry with us. **(2.3)**

To have one of the TAs or someone else teach machine learning, X's lectures were difficult to understand **(2.4)** and the video lengths were too short. It is better to have one continuous video of 30 minutes than 10 videos 2-5 minutes long each **(2.1)**

Either only prerecorded lecture or only the book as a reference. **(3.2)** Sometimes there is more information in the book but it is not clear if we should know this or not. **(2.3)** X's prerecorded lectures are really hard to understand and the information is scattered. The recordings should be done again. **(2.1)**

PLEASE, and with my full respect: help Pr X make clearer videos, those were completly incomprehensible **(2.1)**

Improve the quality of the recorded lectures, especially for the second half of the course. Would be a good idea to remake them with better slides and clearer speech, or do not use them at all and give links to useful materials elsewhere; **(2.1)** Make clear grading scheme and instructions for the written exam. **(4.3)**

While the labs were fun, I would add a few more self-directed tasks to them. Some had them, like when we made our on plots or coloured the proteins in Pymol. I mean similar things for some more labs. **(5.1)**

The sequence report should either have fewer mandatory aspects or a longer word count. It's hard to write a high quality report when there is barely any space for proper discussion or analysis. **(4.1)**

X's videos had very poor audio quality and not very informative slides, this could be improve **(2.1)** Also, I believe that requiring students to have their microphones and cameras on for an exam is against swedish law… **(4.1)**

The book for reading is from years ago. we need something new **(3.3)**

Having better slides for the machine learning section. **(3.3)**

Avoid discussions and do on demand lectures on zoom/ in class **(3.2)**

The second part of the course was a bit challenging to comprehend. Better pre-recorded lectures for some of these topics would be appreciated. **(2.1)**

See q.1

This is a really fast-paced course, especially for those beginners in bioinformatics. Some topics like machine learning and AlphaFold are cutting-edge topics but also difficult for students from biology backgrounds **(2.2)**. I think it will be much helpful if the material used in the machine learning and AlphaFold module are more selective and beginner-friendly. **(3.3)**

More up to date book. I was constantly asking myself if learning these processes in that much detail as written in the book was worth it, since a lot of tools have been preceded by other algorithms. Still I think learning the previous techniques are valuable, but it may be excessive to look at them in such a detail as presented in the book. And in the lecture we barely talk about some of these old techniques. The book is more than 15 years old! and its on Bioinformatics! It is essential I think for this course to get a new course book. **(3.3)** New update on X's recorded lectures. It is not comprehensible what he is talking about, not even to the english first language speakers in the course I talked to. Especially if you hear all this concepts for the first time. **(2.1)**

The new lecture topics were important (AlphaFold, NN), but could use an more general introduction. The TA responsible for the machine learning lab did a great job in finding relevant and understandable analogies. X's lectures were hard to follow. **(2.4)**

Clearer questions on the exam. **(4.2)** Clear exam assessment rules. **(4.3)**

Definitely improve the exam questions. **(4.2)** Another thing is X's prerecorded lectures on machine learning. Unfortunately it was extremely difficult to understand his speaking, perhaps they could be re-recorded and he could speak more slowly and clearly. **(2.1)**

Better prepared lecture material. The audio of Xs lectures were bad, and I did not understand it **(2.1)**

The latter lessons on alphafold and AI felt very disorganised and unfocused. **(2.4)** The examination and grading on them also felt somewhat arbitrary **(4.2)**

include more information in the lectures, e.g. in the RNA structure prediction lecture the topic regarding terms that stabilise / destabilise a structure (among other topics) were not discussed in detail **(2.3)** but in the exam questions were asked regarding that particular topic **(4.2)**

**Course Evaluation 2023**

to broaden aspects of bioinformatics as this was primarily based on structural bioinformatics. **(2.2)**

NO

PyMol can be difficult to navigate at first. Sometimes I felt like I was following instructions without understanding what I was doing. Also, if it's going to be self-taught, I would suggest more guidance in some of the lectures. Sometimes I had to complete the information with a lot of other material and I never knew if maybe I was overdoing it. **(2.3)**

More consideration put into flipped classroms/ change the lecture style; **(3.2)** Less material **(1.2)** and less quizes; **(1.3)** More relevant examination **(4.2)**

The teacher could do a summary at the beginning of each discussion, so it is clear what we were supposed to understand. This is especially important for the second half of the lectures, which dealt with AlphaFold and Machine Learning, as they were not covered in the course book. **(2.3)**

Some recorded lectures were very poor quality which meant a lot more work for the students. **(2.1)**

Maybe it could either be a few less quizzes, **(1.3)** or a few less topics, **(1.2)** or the programming part could be a bit easier . It seemed quite a lot to manage everything, at least for me**.(5.3)**

The quizzes everyday were quite stressful: although they did "force us" to learn, having the lab, the pre-discussion quiz, thediscussion and having to watch the lectures for the next day was a bit too much and I would sometimes finish very late in the day. It would be better if we had more time to learn everything we had during this course **(1.3)** Even though some lectures were really good, some others (Protein-Protein docking and Visualization) were not very clear **(2.4)** and I feel like way too much more information than needed was on the PowerPoint . The book was not up to date for the topic of bioinformatics, unfortunately. **(3.3)** The word limit for the secret sequence exercise was way too small. I genuinely fail to see how it would be possible to get fullmarks with such a low word count. **(4.1)**

I have two suggestions: 1. Rearranging canvas (not having things listed per week and having the same titles for the quizzes as for the lectures) so that everything is searchable and accessible from the home page including schedule and deadlines. 2. Posting learning outcomes with each "kursmoment" so it is clear what the important points to study for the exam are andwhat level we need to know stuff on. Especially for things like basic math and chemistry so we have the opportunity to brush up on it beforehand. **(3.1)**

Y should teach more and X less. Y is generally a better lecturer in all aspects out of all of the lecturers that we had, so maybe give him more lectures to teach! **(2.4)**

Clearly this course is hectic and there is not enough time for assignments. **(1.3)** If possible, it would be great if the deadline couldbe a bit later for the lab quizzes because I barely had time to finish them after the daily discussion sessions. **(1.4)** Some of the labquiz questions were missing important details so they need to be improved. **(5.1)**

for the lectures, **(2)**

I would enhance the communication between teachers and students. I understand teachers are really busy and occuppied, but students should be able to contact them and ask them. This is possible, but not realistically done. **(2.4)**

First of all, examination was unfair. **(4)** Yes, teachers showed us some things how to calculate but teachers didn't spend too much time on that, and it wasn't explained to the details. **(2.3)** Students spent to much hours in doing all kind of quizzes and video lectures that we needed to do before "discussion" part. Even if you give us videos to watch, and not the proper way of teaching (in person) they are not good videos (except phylogeny) **(2.1)**. Secret sequence was too harsh graded and our work andtime wasn't appreciated. Professors and everybody expected from us to teach ourselves (cause we had videos and quizzes) bioinformatics in one month and yet teachers and TA's were to strict in grading our work. Such a shame. Pretty sad. **(4.3)**

**Course Evaluation 2024**

The flipped classroom did not feel well tailored for this course, especially X's recorded lecture felt very unorganized and overall were hard to understand, which made this already dense course even more difficult to properly follow. The course discussions in the morning also felt underwhelming, as they mostly consisted in repeating information that was already available in the pre-lecture material and not really in clearing up unclear notions. **(2.4)** The programming Project, even though it was appropriate in applying the taught skills, did also feel very messy, what we were supposed to do was not clear, and the goal of the project seemed to change mid-project, from constructing a whole model pipeline, to optimizing Z's code, and this one week into the project. It could have been good to have more than one help session, as the project also did require us techniques that were not really seen in lectures or labs. **(3.1)** The exam also did not feel like it was tailored to test understood notions, but moreso just about spitting out the course material without any reflection being asked from the student. Thus, the goal with it felt less like trying to memorize and understand the material, and more about printing as much material for the exam as possible in chance of covering all of theexam's questions. **(4.2)**

I think that the materials covered in the course are too broad, either focus only on ML or more on the theory/proteins part. In my opinion it felt like it was too much information for the length of the course. **(1.2)** it also felt like it was expected that you knew how to program, even though some have no background in bioinformatics/programming and our "introduction" coursein programming was intense and only 4 weeks, which is not enough (in my opinion) to learn how to code such complicated programs by ourselves as we were expected to do in this course. **(5.3)**

The flipped classroom completely didn't work out, and I think they got the concept of flipped classroom wrong. Flipped classroom can work, but not in the way as was done in this course. You cannot expect students to learn everything from just slides without a lecture or good book. This meant that a lot of time was spent looking for good sources to actually learn the material (although P' slides with notes worked reasonably well) **(3.2)**. X just read the slides in his videos which is as good as not having a video. **(2.1)** The labs took a very long time sometimes, even unreasonable to my opinion. **(1.4)** The pre-discussion quiz is great, but make them not graded. This way you just do it to get a good grade which both makes you spent too much time on it, and it won't be informative for the lecturer. **(4.1)**

concept about deep learning **(2.2)**

Have a proper lecture in all parts of the course or a very good video lecture. The YouTube were not good as they didn't have enough information and also the sound quality was not ideal. **(2.1)** Maybe also consider to include a script so students are sure that they understand everything. **(3.3)** Also, increase a bit the report size. In the next course we have reports that are 3000 to 5000. In bioinformatics it could be between 2000-3000. Finally, the labs should be for learning and not an examination. **(4.1)**

As we've already discussed, the number of quizzes for pre-class prep should not be excessive, **(1.3)** and the difficulty of the lab and the number of questions need to be reasonably organized **(5.1)**. 5 days' course a week is a bit too heavy, so 3 to 4 lectures aweek and the rest of the time left for self-study is more reasonable. **(1.4)**

Please come back to normal teaching, due to the knowledge of the course is not suitable for self-study **(3.2)** Please consider deeply the assignments/quizzes and its deadlines, it is too much for a day to handle everything with proper knowledge learned. **(1.3)** I would suggest setting the deadline for all after-quizzes to the end of the course, i.e. the student can choose when to finish them, to get their own study pace. If you want to let students learn by themselves, they should be allowed to learn at their own pace. **(3.2)** Please be responsive to students' feedback. Please provide feedback for marks lost in assignments. **(3.1)**

Flipped classroom. The idea is good but is hurt by the execution. The lecture material handed out is difficult to grasp for different reasons. P lecture slides were ok, but as evident from the discussion, he is a great lecturer, and having him hold the lecture would have been much more engaging. **(3.2)** X’s videos were largely incomprehensible, and it was difficult to discern what was important info and what was editorial commentary. **(2.1)** To adress this a list of key concepts should have been handed out. The AI videos by Z had a good structure, but having him read a very info-dense script meant that I had to constantly pause and rewind to capture all the intricacies. This could be resolved by having Z actually do a lecture, and thereby reasoning through the concepts at a comprehensible pace. **(2.4)**

Replace the morning discussion sessions with actual lectures. Without them we could not just learn the material properly and figuring it out at night by ourselves was too labor- and time-intensive. **(3.2)** Then the 50 page readings per day also become supplementary because we would be explained the materials in the morning. This decreases the workload dramatically and allows us to actually critically engage with content while keeping our health and mood stable. **(1.3)** The labs were ok and can be kept except that you need to make sure they do not take longer than they are scheduled for (and often they did) **(1.4).** Make sureall the quizzes are correct because there are far too many mistakes in them. It was not even funny when we kept finding confusing mistakes over and over. It was not always the case but still more often than it should be. It is lousy and sloppy job on the end of the course coordinator. **(4.2)**

- at least one day off per week **(1.4)** - pre-quiz scores should have not been counted for the final grade - **(4.3)** lab quizzes should take no longer than 2 hours (the last ones were better) **(1.4)** - the preparation material was useless to correctly answers the pre-quiz questions **(4.2)** - the first discussions we had were bad: it does not matter what was the most difficult/cool thing of the provided material if it is not further explained during the discussion **(2.4)**- the exam should have been earlier to give us more time to work on the assignment - some questions of the exam were of questionable utility: is copying the picture of the structure of Evoformer actually assessing my knowledge better than the description of it that I provided?! **(4.2)**

Considering what I said above, I would change a lot in this course, in fact maybe everything. I would make the lectures real lectures and not just discussions. I would alter the lab quizzes - exercises to learn the use of important tools should be done before one is evaluated on that content - that is, one should not be evaluated in something while you are learning it for the 1st time. **(3.2)** I would adapt the assignment expectations and prepare the students a bit more for it - including perhaps more specific directions **(2.3)** and a bigger size report so the student actually has the words to go into what was commented one should have explained. **(4.1)** Finally, I would make the exam format different to evaluate the knowledge more reasonably - for example, increase the number of questions and make each one more direct to a point. **(4.2)**
