# Supplementary material for: Combining flipped-classroom and spaced-repetition learning in a master-level bioinformatics course
Source: PLoS Comput Biol. 2025 Apr 15;21(4):e1012863. doi: 10.1371/journal.pcbi.1012863 (PMC11999146; doi:10.1371/journal.pcbi.1012863)
Supplement: S5 Appendix — (DOCX) [file pcbi.1012863.s005.docx]

QUAGOL STAGE7: coding

1 Learning Rate (time to acquired knowledge ratio)

2 Teaching

2.1 Topics (appreciation of one or all topics)

2.2 Pedagogical skill (individual teachers’ skill)

3 Course organization

3.1 Structure (course management and methods)

3.2 Materials (quality and availability of book, slides and video)

4 Examinations (Project & Exams)

5 Laboratories

5.1 Quality (appreciation of laboratory activities)

5.2 TA Support (appreciation of TA interactions)

5.3 Programming (investment in programming)

**Course Evaluation 2018 (19 answers)**

I like the possibility to learn programining. **(5.3)**

The lab part **(5.1)**

good help in the labs **(5.2)**

That I learn things about programming I did not know before. **(5.3)**

The curriculum included some good background basics. **(2.1)**

1. All python labs **(5.3)**

2. Machine learning related lab and lecture **(2.1)**

3. Phylogeny related lab and lecture **(2.1)**

That I learnt the basics of Python programming, **(5.3)** as well as some aspects of bioinformatics. **(2.1)**

The understanding and student-friendly approach on the course from Y. **(2.2)**

The subject **(2.1)** and computer labs **(5.1)**

being forced to learn python **(5.3)** in a short time **(1)** lectures of Y and G **(2.2)**

The firm foundation laid by lab courses.**(5.1)**

The study material which was provided. **(3.2)**

It was quite intensive **(1)** and helped me to get a good knowledge of coding**(5.3)** and bioinformatics**(2.1)**

That we were introduced to python programming (and it was half beaten into us) **(5.3)** and how to use tools in bioinformatics. **(2.1)** That it ended.

programming part **(5.3)**

The subject. I like bioinformatics, **(2.1)** even though I thought this course was bad.

Some of the lectures were really good (I liked P’s and Y's lectures). Actually the lectures in general were enjoyable and nice, **(2.2)** it's just that they were completely irrelevant in the context of the assignment hell we were going through. I also very much like the fact that the course has it's own webpage, a schedule and all tasks at one place. That is very good. **(3.1)**

The practical approach to programming including the python sessions **(5.3)**

**Course Evaluation 2019 (23 answers)**

The quizzes for each lecture **(3.1)** and lab **(5.1)**.

In general the content, which was very interesting and relevant. **(2.1)** More specifically, I enjoyed the labs. **(5.1)**

The amount of mainly new information conveyed. **(1)** The computer labs and that there we got to use the knew skills. **(5.1)**

The pre-lecture reading **(3.2)** and quizzes helped me understand the lecture deeper to a large degree. **(3.1)**

Lectures and practical excersises were well combined. **(3.1)**

The computer labs **(5.1)** and the secret sequence project. **(4.1)**

The secret sequence report for me was the most (if not the onnly) fun part of the course that I learned a lot of things and I used all the provided knowledge. I wish we had more time to prepare it to be able to go more in depth in our assignment. **(4.1)**

Learning the theory behind widely used approaches, such as hidden markov models, neural networks, and machine learning. Learning how to use widely used databases and tools in bioinformatics. **(2.1)**Gaining more practice with terminal and working with code. **(5.3)** Also found the homology modeling very exciting. In general, learning the theory behind bioinformatics **(2.1)** and then applying the learning in the labs, the application part was the best part of the course. **(5.1)** I learned a lot during the course. **(1)** The reading was very helpful to understand the material. **(3.2)**

The content is so useful. I did not expect that we can hand on with many tools in every aspect of bioinformatics. **(1)**

Interesting course content, **(2.1)** and being able to implement new knowledge during the practical lab sessions.**(5.1)** Also, the secret sequence report provided a nice opportunity to learn about databases & different methods to apply when studying an unknown sequence.**(4.1)**

The variety **(1)**

- Lab **(5.1)** and pre-lecture quizzes for application and deeper understanding of the aspects in the lectures - moreover it was so nice that the quizzes could be repeated, cause you had the chance to improve your points/grade in the end my reading and studying again/found out more aspects **(3.1)** - it was possible to ask every question in everytime and all people (docents **(2.2)** or TAs) were very helpful, really tried to show and explain everything that I really understood the important aspects **(5.2)** - all lectures were recorded --> learning and repeating of aspects in the lecture were possible in detail **(3.2)** - the exam was splitted in two parts **(4.1)**

Y lectures were really clear and interesting. **(2.2)**

The lab crew. I would be completely lost without them, and somehow they made me not leaving the course. **(5.2)**

The labs were the best part of the course, by putting in practice some of the theory is how I best understood the concepts and helped me in the study for the exam **(5.1)**

I would have liked to learn how to use a program. The teachers of the labs. **(5.2)**

The first section of the course where the learning was oriented to the prediction methods and theory related to the sequence aspect was very helpful and useful for the practical purposes as a student. **(2.1)**The Labs were equally useful and helpful in developing deeper understanding about various topics. **(5.1)** Last but not the least the elaborate discussions **(3.1)** and the time devoted in the class and in the breaks by the professor ensured that the concepts were crystal clear **(2.2)**

prequizz, it is really helpful **(3.1)** the record is also useful especially for review **(3.2)**

Molecular modeling, **(2.1)** the lab sessions always fun and exciting as we can try. **(5.1)**

Application and lab practice to exercise various bioinformatic tools. **(5.1)**

I think that was the course from which I have gained the most knowledge since I started this master's degree. **(1)** I found the content of this course excellent. **(2.1)**

Labs, **(5.1)** Secret Sequence**(4.1)**

**Course Evaluation 2020 (30 answers)**

The format **(3.1)**

The course is very well structured and the learning goals are very clear. **(3.1)** The evaluation is also very explicit and fair, unlike previous courses where several assignments were irrelevant for the final grade. (4.3) The topics discussed are extremely relevant and helpful.**(2.1)** Professor Y is very helpful, **(2.2)** as well as the TA's S and E, which are always happy to help with any Question. **(5.2)**

-The guest lectures were really good and informative **(2.2)**

-The labs are a nice way of incorporating the gained knowledge and applying it. The secret sequence labs were also nice to allow and enable us to work on our report. **(5.1)**

-Filming of the lectures, which made studying easier (if you miss something, you can listen to the explanation after class **(3.2)**

Learning about **(2.2)** and using several different bioinformatics tools, learning some programming basics.**(5.3)** Teaching staff was open to adjust the workload based on the feedback from students.**(2.2)** Cake

The help of the TAs during the labs.**(5.2)**

Helpful TA's that introduced the topics on a level that was easy to udnerstand for those who did not have any previous knowledge within this field.**(5.2)**

- Labs**(5.1)**

- secret sequence report**(4.1)**

Most of the labs were really nice and I enjoyed the application of the learned databases**(5.1)**

Good over all knowledge about Bioinformatics **(1)**

the practical lab**(5.1)**

Lectures were recorded and uploaded to youtube (I never could open the lectures in .MOV format though)**(3.2)**

Recorded lectures!**(3.2)**

Tools taught in this course are absolutely vital for any biochemist. **(2.1)**

The lab was great to Practice.**(5.1)**

The lectures**(2.2)** and labs were quite informative.**(5.1)**

Both lectures**(2.2)** and labs.**(5.1)** The labs we have many good TAs that could help to solve problems patiently. **(5.2)**The lectures have the record.**(3.2)** Pre- lecture quiz could push students to read actually.**(3.1)**Unix and python are interesting.**(5.3)**

combination if lecture material and labs. moreover, the secret sequence report in the end allowed application of everything learned one more time.**(3.1)**

Labs**(5.1)**

The labs were a really great learning experience. They were well structured and executed quite well.**(5.1)** That in addition with the secret sequence assignment ensured practical application of all the tools and approaches learnt during the course.**(4.1)**

the combination of lecture and lab**(3.1)**

Lab sections.**(5.1)**

The lab work **(5.1)**and continuous evaluation.**(3.1)**

I think the labs were the best part of the course.**(5.1)**

All the skills we acquired at the labs**(5.1)** and the chance to use the for the secret sequence report. **(4.1)**All assistants in the lab were really helpful and good at their job.**(5.2)**

Interactive computerlab sessions**(5.1)** with a lot of help from the teaching assistants.**(5.2)**

The labs**(5.1)** and TAs.**(5.2)**

That there was so much practical work, including programming. And that many of the tutors were amazing (I'm thinking mainly of Eloy and Sarah). I also appreciated that there were so many tutors during the practical work, and that they helped me to not feel stupid at the times when I was struggling with programming. Extremely appreciated.**(5.2)**

The video recording of every lecture was pretty useful.**(3.2)**

The course covered many important areas of bioinformatics. **(1)** The best part of the course was the secret sequence report. It was fun but I regret that I didn't get enough time to work on it(because of personal reasons). I had to submit it in hurry. **(4.1)**The system of video lectures was great. All courses should have that. It helps students a lot because we all have different speed of grasping things in the class. So recorded video lectures are a valuable resource.**(3.2)**

The lab practice,**(5.1)** with the use of scripts. It really felt like the theory was being applied, and let us look into things I never imagined how to code like neural networks.**(5.3)** Secret sequence was also a very neat homework. Having 2 partial exams instead of a big one is also good, especially with all the work we have during the course. **(4.1)**

**Course Evaluation 2021 (29 answers)**

Coding**(5.3)**

It has a lot of condensed material in little time, so it forces one to learn fast, and I felt that I really learned a lot**(1)**

Combination of pre-recorded lectures, discussions and practical computer labs.**(3.1)**

Secret sequence report**(4.1)** and the labs**(5.1)**

The course is well organized **(3.1)**and the TAs are very helpful.**(5.2)**

Learning bash and python will be very useful for me in the future.**(5.3)** I also found the secret sequence report highly useful for practicing everything we learned during the course.**(4.1)**

The labs **(5.1)**and the associate teachers. They were fantastic**(5.2)**

It was interesting **(2.1)**

The structure was very good and it was easy to know what was expected of us. The lectures being on youtube was also very nice.**(3.2)**

The best and the worst part of this course was the amount of new notions we have learnt. It was very satisfying at the end of the course knowing how many new concepts we understood.**(1)** The Labs were the best part of the learning process since based on the pratical use of tools that we're gonna use in our research future. **(5.1)**

the labs **(5.1)**and helpful TAs**(5.2)**

DEFINITELY the labs, it felt very hands-on and they were useful for our understanding.**(5.1)** Also, I liked that the lectures were recorded so we could study at our own pace (I hate live Zoom lectures).**(3.2)**

The labs were quite usefull for understanding an practicing on the theoreticall content**(5.1)**

The concepts were relevant **(2.1)**

The labs were pretty useful**(5.1)** but personally, I really liked the Secret sequence assignment we literally applied all the knowledge gathered during the course especially at the labs and use it to "characterized" a protein.**(4.1)** the TAs were also awesome really involved in the class' dynamics and always open to help you out.**(5.2)** The sessions with Maria Bueno help me to speak out about small doubts that probably were not worthy to comment in the big group and go over the topics together drawn a big picture of the content. She led us really well!**(3.1)**

Computer labs and exercises.**(5.1)** Also, I liked the flipped classroom approach.**(3.1)**

The various tools that are used to visualize Proteins, DNA and RNA. **(2.1)** How well one can get so much of information from a given known or unknown sequence.**(1)** It was a great experience to understand **(2.1)** and also work on these tools.**(5.1)**

The inverted classroom was really great! I also really liked that we came across the topics several times. 1. In the book 2. In the lecture based on the book chapter 3. In the test, to make us think about, what we just read. 4. In the inverted classroom Zoom Sessions. 5. In most labs/lab quizzes, to make us think again :) I also liked the good structure that was eazy to follow.**(3.1)** The knowledge gained was also very good, but Python was kinda "squeezed in" and was too much.**(2.1)**

Flipped classroom organised that way really worked for me.**(3.1)** And the project was a cool thing to do.**(4.1)**

1.Quizzes which motivated students to be prepared before each session.**(3.1)**

2. The TAs were great. It was very nice to work with all of them during the lab-sessions. They were very helpful and clear with the explanations**(5.2)**

the help we received from the previous students was really helpfull.**(3.1)**

It was great how many TA's there were and how responsive they were. Overall i found the environment to be high support. This was especially true of the labs and it was really nice having them on zoom with different rooms where we could collaborate and ask the TA questions.**(5.2)**

Content **(2.1)** and flexibility**(3.1)**

I would say the project part.**(4.1)**

Best aspect was that it was practical, I liked the labs (most of it), because thats how I get valuable experience.**(5.1)**

The combination of labs with discussion sessions.**(3.1)** I think it was great to learn and apply the knowledge straight after, I felt it helped a lot to understand the concepts!**(5.1)** Also, I liked to have labs in an online Zoom format. It felt very easy to reach to TAs, who were always there for you.**(5.2)**

**Course Evaluation 2022 (26 answers)**

Y‘s lectures.**(2.2)**

In general, I really liked the structure of the course. In the beginning it might seem like a lot but it’s pretty educative and really fun to learn.**(3.1)** TAs are also very nice and helpful.**(5.2)**

The up-to-date technology**(3.1)**

The labs. Definitely the effort to takes us on a not too overwhelming learning journey with the practical exercises was noticed.**(5.1)** M's lecture on phylogenetics was also very clear, organized and to the point. Informative enough and very well presented. P's discussion session was also good. Good guidance around very specific questions (without letting too much up to the students how to run the session, like the other sessions) helped us to stay focused on the most important topics.**(2.2)**

The labs**(5.1)**

see the same topic for preparation, discussion and lab. It enables a better assimilation of the matter.**(3.1)** The labs were well made.**(5.1)**

very good introduction to many bioinformatic concepts, don't reduce the number of subjects**(1)**

- Interesting topics;**(2.1)**

- Despite online teaching, the course is very interactive and try to make students feel involved;**(3.1)** Well-designed lab sessions**(5.1)** and good TAs-**(5.2)**

I liked the creative/self directed aspect of the sequence report.**(4.1)** I also enjoyed the labs very much, all of them were high quality.**(5.1)**I loved the teaching style with the pre-recorded lectures**(3.2)**, quizzes and discussion section. It was easy to keep up. **(3.1)**

learning the databases and tools during the labs,**(5.1)** and writing the secret sequence report.**(4.1)**

The labs were the best part, it is were we actually got to do stuff.**(5.1)**

The labs,**(5.1)** challenging topics**(2.1)**

The resources for studying the course,**(3.2)** as well as the labs, were excellent. The labs were conducted in a very efficient way**(5.1)** and the flipped classroom pattern of studying was very beneficial.**(3.1)**

I really liked the labs, they helped understand the material in a different way**(5.1)**

The lab is beneficial for me, giving us some hands-on opportunities to perform programming and basic bioinformatics analysis.**(5.3)**

Getting to know the

The final report really tied in all the knowledge we acquired during the course. Showed how much we learned in a month. **(4.1)** Loved the flipped classroom approach.**(3.1)** The labs were very well structured,**(5.1)** the TAs were super helpful. It worked really well even through zoom.**(5.2)**

The concept of the flipped classroom. It was convenient and easy to follow.**(3.1)** In addition, the presence of the coursebook and reading materials were useful.**(3.2)**

The format was great. It was definitely a challenging course that required a lot of studying, but i liked the prerecorded lectures + reading followed by discussions and labs.**(3.1)** I definitely learned a lot this course.**(1)**

The Labs**(5.1)**

Experienced wide range of bioinformatics tools for practical use in the future**(1)**

Learning practical tools such as pymol**(2.1)**

the labs were helpful in further understanding of the lecture materials**(5.1)**

The help of TA's**(5.2)** and the course structure**(3.1)**

**Course Evaluation 2023 (14 answers)**

learning about the various bioinformatic tools**(2.1)**

the lecture video **(3.2)** and the open-book exam

It was very much the perfect level for me. I had some notion of bioinformatics, but it was quite limited. This gave me so much new information and material. I very much enjoyed the learning.**(1)**

The labs **(5.1)**and lab help**(5.2)**, secret sequence report was good for reinforcing the learning-outcomes. **(4.1)** The lecturers were mostly open to engage with students wich was good and created a safe enviornment for questions**(2.2)**

The labs, as they allowed us to learn how to practically use several biological databases.**(5.1)**

The quizzes really pushed me to study the whole time which was good.**(3.1)**

The prerecorded lectures made it easy to go back and re-listen to the topics that might seem more difficult. **(3.2)** Most of the quizzes were fun to do and helped to understand the concepts, however the workload seemed to be too big, since e.g. programming quizzes seemed to be more advanced than our level of programming.**(5.3)**

Learning about many different topics.**(1)** Some lectures were really good, especially those on heuristic search methods, and RNA structure determination, profiles and HMMs, phylogeny...**(2.2)**

I learnt a lot of interesting things that can be useful for us the future.**(1)** I also thought the quizzes were really helpful.**(3.1)** The lecturers were great, and I appreciated getting the opportunity to ask questions and discuss answers too.**(2.2)**

The labs were great!**(5.1)**

All the new and exciting topics I got to learn about. I think the course has great content **(2.1)** and the professors/TAs, especially Y and G, were really good!**(2.2)**

the labs**(5.1)**

The theory that was given**(2.1)** and how it was given.**(2.2)**

None

**Course Evaluation 2024 (12 answers)**

The chosen topics were very relevant as part of a researcher's toolkit.**(2.1)** Labs also were relatively well made, even if sometime a bit too long, and tested and required us to apply important notions from the lectures.**(5.1)**

The TAs were very helpful**(5.2)** and the recorded lectures by Z were good.**(3.2)**

The neural network part, and 'learning' how to make them (although most of it I learned by myself).**(2.1)**

deep learning study**(2.1)**

Neural Networks by far. **(2.1)** Really concise,**(2.2)** nice labs**(5.1)**, nice assignment. A bit bigger report would be nice so we can explain morewhat we did because some of us did quite a lot of things that we have to skip in the report. **(4.1)** Other than that this part of the course was really informative, helpful and nice.**(2.1)**

The content of the course is reasonable, the neural network part is difficult but necessary,**(2.1)** and the collection of labs**(5.1)** and lectures can help to better understand the knowledge points of the lectures.**(2.2)**

Neural network lectures are very good**(2.2)**

Interesting content. **(2.1)** The AI lectures made the subject interesting and understandable.**(2.2)**

The topic on which the course is.**(2.1)** The materials that we were supposed to learn were actually great.**(3.2)** If only the course gave space for actually learning them it would be great.

The content was interesting and useful**(2.1)**

The best part, if I had to say one, would be the deep-learning section of the course where I finally felt we were actually receiving teaching - Z was an attentive lecturer who always looked to answer our questions and was understanding of the fact that we had a limited and tight schedule to learn such an intense amount of concepts - concepts indeed important but that need time and could compose a course for themselves.**(2.2)**
