# Supplementary material for: Combining flipped-classroom and spaced-repetition learning in a master-level bioinformatics course
Source: PLoS Comput Biol. 2025 Apr 15;21(4):e1012863. doi: 10.1371/journal.pcbi.1012863 (PMC11999146; doi:10.1371/journal.pcbi.1012863)
Supplement: S6 Appendix — (PDF) [file pcbi.1012863.s006.pdf]

Secondary Structure Prediction

Introduction

Protein secondary structure prediction is a crucial task in bioinformatics, aiming to determine the local structural elements of proteins. Traditional methods rely on experimental techniques like X-ray crystallography and NMR spectroscopy, which are time-consuming and costly. In recent years, neural networks have shown promise in predicting secondary structures from amino acid sequences [1]. By utilizing multiple sequence alignment data, these networks can capture evolutionary information to enhance prediction accuracy [2]. The state-of-the-art methods include deep learning architectures such as recurrent neural networks (RNNs) and convolutional neural networks (CNNs), which have demonstrated superior performance in classifying secondary structures into helix, strand, and coil classes [3]. The 3 classes are illustrated in Figure 1. This paper aims to explore the effectiveness of fully connected neural networks, utilising the sliding-window method, in improving the accuracy and efficiency of protein secondary structure prediction.

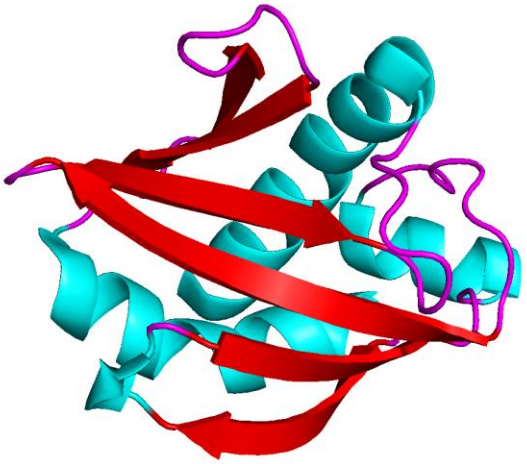

Figure 1. Secondary structure elements, α-helix (blue), β-sheet (red) and coil (pink).

Materials & Methods

Data

The training and test datasets utilized in this study were sourced from Katarina Elez's GitHub repository, available at https://github.com/katarinaelez/protein-ss-pred.git. These datasets comprise Multiple Sequence Alignment files, from which input features such as Position Specific Scoring Matrices (PSSM) and amino acid sequences were extracted. Additionally, the datasets include files containing the output labels, representing the 3-class secondary structures.

A sliding-window method was applied to both features to create profiles. This method involves iterating over each residue in the protein sequence and extracting a window of neighbouring residues centred around it [4]. The output labels were numerically encoded to represent the three

classes: 0-Helix (H), 1-Strand (E), and 2-Coil (C). The amino acid sequence profiles and output labels underwent one-hot encoding to ensure that all input features and output labels are in a suitable format. Finally, predefined cross-validation sets were created to ensure the development of a robust model.

Model Development

We tested various combinations of optimizers (SGD, Adam) and activation functions (tanh, ReLu, Leaky-ReLu), explore different numbers of hidden layers and units per layer, and evaluate regularization methods such as dropout and activity regularization (L1 and L2). This comprehensive exploration aimed to identify optimal configurations for enhancing model performance while mitigating overfitting. The hyperparameters of the final model were further optimized using the Bayesian optimizer, a sophisticated algorithm that efficiently explores the hyperparameter space by leveraging probabilistic models to guide the search towards promising regions.

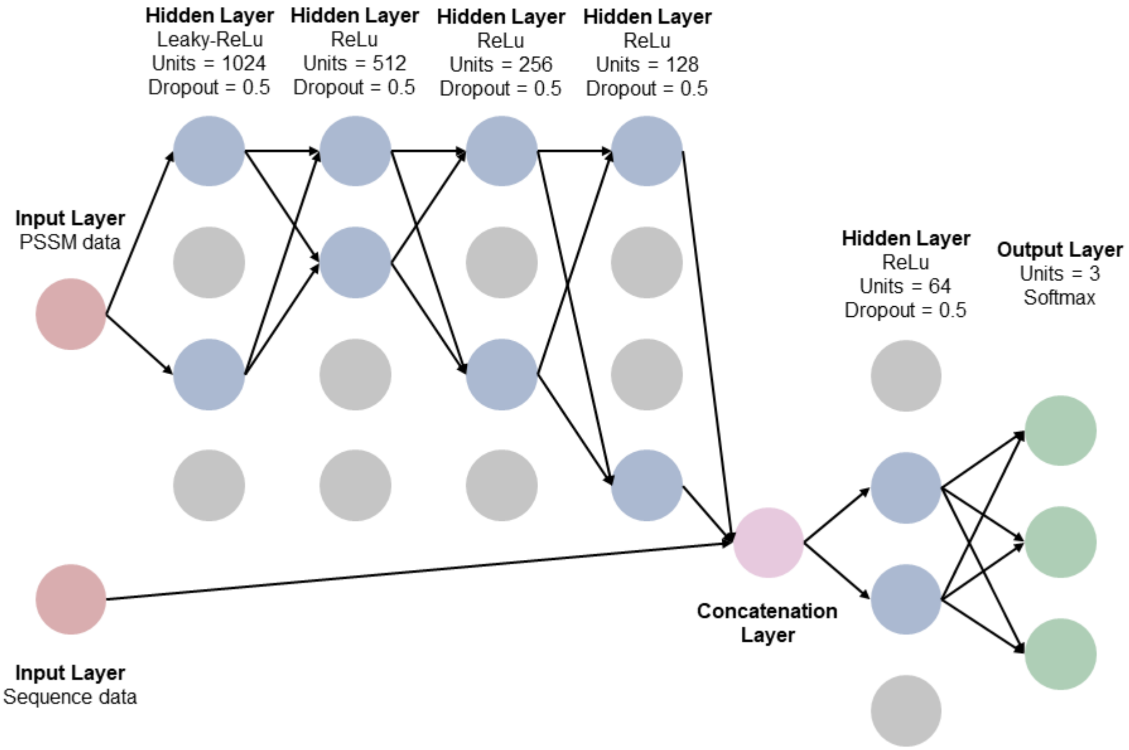

Figure 2. Schematic Illustration of ensemble model architecture.

The final ensemble model architecture is schematically illustrated in Figure 2 and comprises two input branches. The first branch processes PSSM profiles through a series of densely connected layers, including four hidden layers with 1024, 512, 256, and 128 units, respectively. Each hidden layer is followed by a dropout layer with a dropout rate of 0.5 to prevent overfitting. Meanwhile, the second branch directly passes amino acid sequence profiles to the output. The outputs of both branches are then concatenated and fed into a dense layer with 64 units and ReLu activation.

This layer is followed by a dropout layer for regularization before the final output layer with three units and SoftMax activation, representing the probabilities of the three classes.

Model Evaluation

For model evaluation, a one-vs-all approach was employed to assess the performance of the 3-class classification. Metrics including accuracy, sensitivity, and specificity were computed individually for each class. Overall accuracy was also calculated. This comprehensive evaluation strategy provided insights into the model's ability to distinguish between different classes and its overall predictive capability.

Results

Figure 3 illustrates the correlation between model accuracy and window size [4], while Figure 4 presents the results of the ablation study, where each boxplot showcases the 5 cross-validation accuracy values for different models. Table 1 displays the accuracy for all cross-validation and blind test sets, along with their respective means. Additionally, Figure 5 depicts the confusion matrix for the best predicted blind set (model trained on cross-validation set 3), while Figure 6 provides insights into the mean accuracy, specificity, and sensitivity per class for the optimized ensemble model.

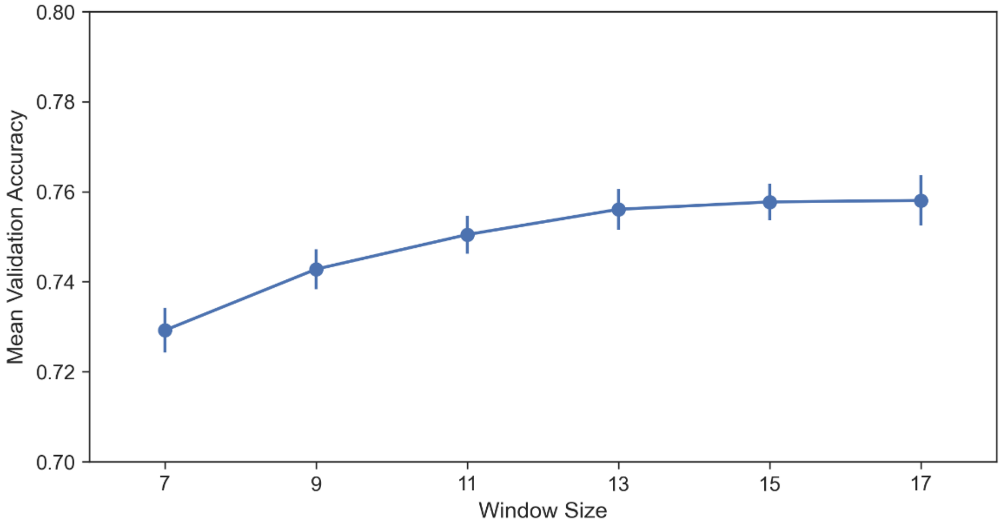

Figure 3. Window size versus mean accuracy of cross-validation sets.

Table 1. Best model's accuracy for cross-validation and blind test sets.

	CV-1	CV-2	CV-3	CV-4	CV-5	Mean
CV Accuracy	0.77	0.7711	0.7603	0.7708	0.7736	0.7692 ± 0.0046
Blind Test Accuracy	0.7516	0.756	0.7595	0.7556	0.7584	0.7562 ± 0.0027

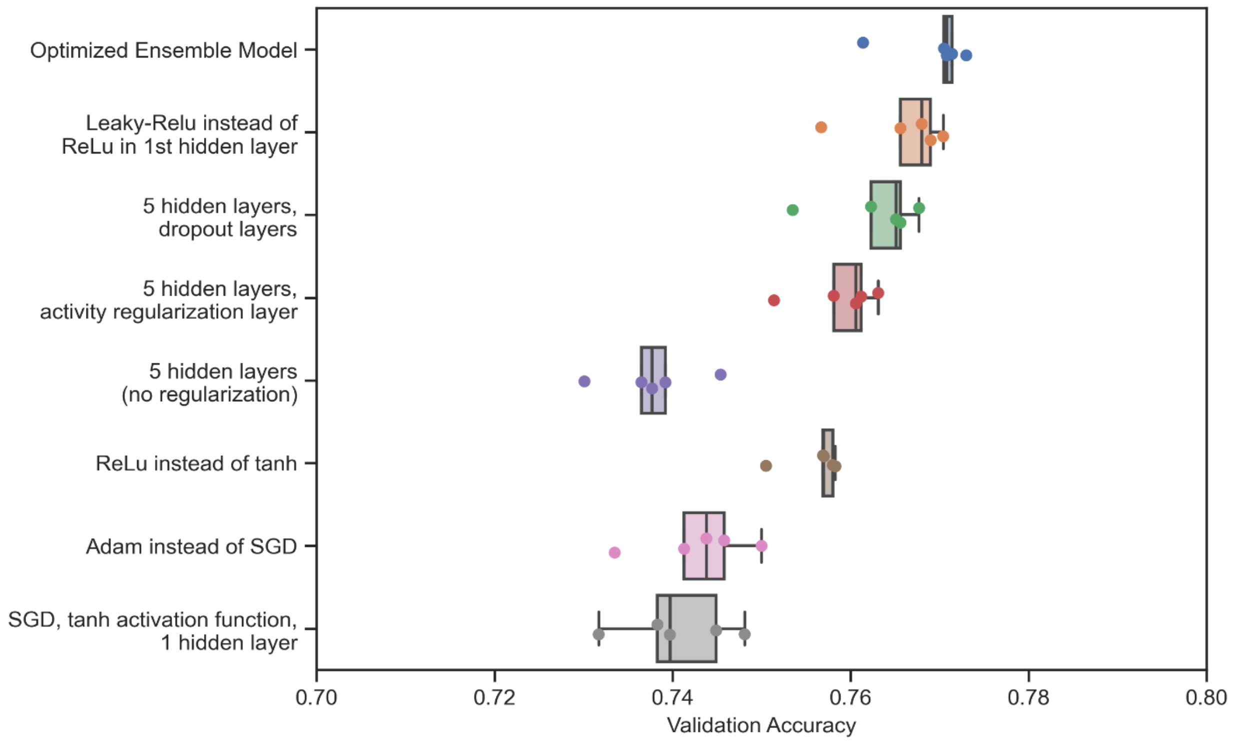

Figure 4. Accuracy of cross-validation sets for different models.

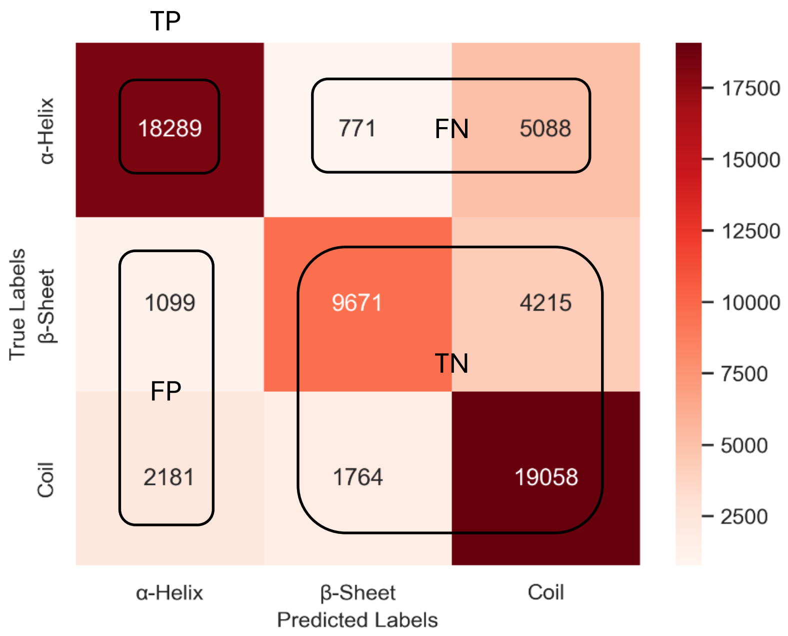

Figure 5. Confusion matrix of the model trained on cross-validation set 3. Annotation of true positives (TP), true negatives (TN), false positives (FP) and false negatives (FN) for α-Helix using one-vs-all approach.

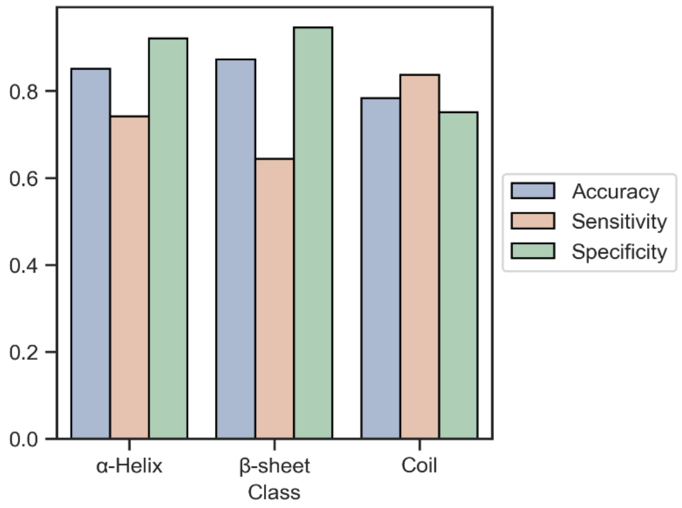

Figure 6. Mean accuracy, sensitivity, and specificity per class.

Discussion

Figure 3 suggests that increasing the window size enhances model accuracy, with a slight improvement observed at size 17, prompting its selection for model training. Notably, beyond window size 13, accuracy appears to plateau. In Figure 4, switching the solver to Adam and using ReLu activation for hidden layers improved model accuracy and addressed vanishing gradient issues, especially as the network became deeper. However, adding layers without regularization led to overfitting, something that was mitigated by introducing activity regularization or dropout layers. Dropout outperformed L1/L2 regularization.

Further enhancement was achieved by adopting Leaky-ReLu activation and incorporating sequence profiles in an ensemble model. Despite a slight decrease in performance on the blind test set showcased in Table 1, the model demonstrates satisfactory generalization. Moreover, Figure 6 highlights β-sheets as the most accurately predicted class, while coils exhibit the lowest accuracy. These metrics were derived from confusion matrices, such as the one illustrated in Figure 5, where True Positives (TP), True Negatives (TN), False Positives (FP), and False Negatives (FN) are annotated, exemplifying the case of α-Helix using a one-vs-all approach.

This comprehensive exploration underscores the critical role of hyperparameter optimization and architectural design in enhancing the accuracy and robustness of protein secondary structure prediction models. By systematically evaluating various configurations and leveraging techniques like dropout and ensemble modelling, we have demonstrated significant improvements in predictive performance. These findings highlight the potential of neural networks for developing more accurate and reliable computational tools for protein structure analysis.

References

[1] D. T. Jones, “Protein Secondary Structure Prediction Based on Position-specific Scoring Matrices”.

[2] S. Wang, J. Peng, J. Ma, and J. Xu, “Protein Secondary Structure Prediction Using Deep Convolutional Neural Fields,” *Sci Rep*, vol. 6, no. 1, p. 18962, Jan. 2016, doi: 10.1038/srep18962.

[3] Y. Zhou, A. Kloczkowski, E. Faraggi, and Y. Yang, Eds., *Prediction of Protein Secondary Structure*, vol. 1484. in Methods in Molecular Biology, vol. 1484. New York, NY: Springer New York, 2017. doi: 10.1007/978-1-4939-6406-2.

[4] K. Chen, L. Kurgan, and J. Ruan, “Optimization of the Sliding Window Size for Protein Structure Prediction,” in *2006 IEEE Symposium on Computational Intelligence and Bioinformatics and Computational Biology*, Toronto, ON, Canada: IEEE, Sep. 2006, pp. 1–7. doi: 10.1109/CIBCB.2006.330959.
