# Supplementary material for: Combining flipped-classroom and spaced-repetition learning in a master-level bioinformatics course
Source: PLoS Comput Biol. 2025 Apr 15;21(4):e1012863. doi: 10.1371/journal.pcbi.1012863 (PMC11999146; doi:10.1371/journal.pcbi.1012863)
Supplement: S7 Appendix — (DOCX) [file pcbi.1012863.s007.docx]

Short questions (8p per question):

1. Describe how ClustalW (and other progressive multiple sequence alignment methods) works?
2. What is the “power law–like distributions of paralogous family size in a genome”? Explain how this describes the different protein family sizes (4p). Which fundamental evolutionary principle might have caused this distribution (4p)?
3. Describe and draw the TMHMM method that is used for membrane protein topology predictions (4p). How is the length of the helices modeled (2p)? What is the “positive inside rule” and how is it taken into account in the model (2p)?
4. (A) How many degrees of freedom (e.g., angles) do you need to describe the backbone conformation of a protein chain of length N residues? (4p) (B) What amino acids does this Ramachandran plot represent (4p)?


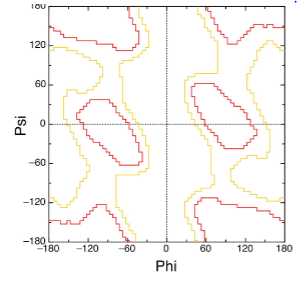


1. Given an identity matrix (score of 1 for matches and 0 for mismatches) and a gap penalty of -1 per gap what is the optimal local alignment and score of the following sequence pairs (4p per pair):

IKLALL / IKAL

WYIKLLLL / WIKLLLK

1. (a) Describe how a supervised machine learning method works (4p)? Describe some factors that are important to obtain the best result. (b) In particular what is overtraining (2p) and (c) how can it be avoided (2p)??
2. What is the difference between a parsimonious and a neighbor joining method in phylogeny ?
3. How is the E-value in sequence alignment dependent on the score and the database size?

Long question (16p):

Describe how you would develop a predictor that is able to identify the interaction areas between two proteins. What methods would you use? What are the important data you can use? How would you develop the pipeline? Describe potential problems. What proteins are easier to predict?
